# SMYD2 lysine methyltransferase regulates leukemia cell growth and regeneration after genotoxic stress

**DOI:** 10.18632/oncotarget.15147

**Published:** 2017-02-06

**Authors:** Adi Zipin-Roitman, Nasma Aqaqe, Muhammad Yassin, Shahar Biechonski, Mariam Amar, Mark F. van Delft, Olga I. Gan, Sean P. McDermott, Alla Buzina, Troy Ketela, Liran Shlush, Stephanie Xie, Veronique Voisin, Jason Moffat, Mark D. Minden, John E. Dick, Michael Milyavsky

**Affiliations:** ^1^ Department of Pathology, Sackler Faculty of Medicine, Tel Aviv University, Tel Aviv, Israel; ^2^ Princess Margaret Cancer Centre, University Health Network, University of Toronto, Toronto, Canada; ^3^ Walter and Eliza Hall Institute of Medical Research, Melbourne, Australia; ^4^ Leidos Biomedical Research, Washington D.C., USA; ^5^ Department of Immunology, Weizmann Institute of Science, Rehovot, Israel; ^6^ Donnelly Centre for Cellular and Biomedical Research, University of Toronto, Toronto, Canada; ^7^ Princess Margaret Cancer Centre, University Health Network, University of Toronto, Toronto, Canada; ^8^ Department of Molecular Genetics, University of Toronto, Toronto, Canada; ^9^ Department of Medicine, University of Toronto, Toronto, Canada

**Keywords:** SMYD2 lysine methyltransferase, AML, quiescence, chemotherapy, DNA damage

## Abstract

The molecular determinants governing escape of Acute Myeloid Leukemia (AML) cells from DNA damaging therapy remain poorly defined and account for therapy failures. To isolate genes responsible for leukemia cells regeneration following multiple challenges with irradiation we performed a genome-wide shRNA screen. Some of the isolated hits are known players in the DNA damage response (e.g. p53, CHK2), whereas other, e.g. SMYD2 lysine methyltransferase (KMT), remains uncharacterized in the AML context. Here we report that SMYD2 knockdown confers relative resistance to human AML cells against multiple classes of DNA damaging agents. Induction of the transient quiescence state upon SMYD2 downregulation correlated with the resistance. We revealed that diminished SMYD2 expression resulted in the upregulation of the related methyltransferase SET7/9, suggesting compensatory relationships. Indeed, pharmacological targeting of SET7/9 with (R)-PFI2 inhibitor preferentially inhibited the growth of cells expressing low levels of SMYD2.

Finally, decreased expression of SMYD2 in AML patients correlated with the reduced sensitivity to therapy and lower probability to achieve complete remission. We propose that the interplay between SMYD2 and SET7/9 levels shifts leukemia cells from growth to quiescence state that is associated with the higher resistance to DNA damaging agents and rationalize SET7/9 pharmacological targeting in AML.

## INTRODUCTION

Acute myeloid leukemia (AML) is a heterogeneous group of blood malignancies characterized by accumulation of malignant blast cells in the patient's bone marrow [1]. AML arises due to deregulation of molecular pathways controlling self-renewal and differentiation processes in the primitive hematopoietic stem and progenitor cells (HSPC) [1]. Despite the recent advances in our understanding of AML genetics and extensive modeling of the disease *in vitro* and *in vivo* the long-term survival remains dismal especially for elder patients [1–3]. Experimental evidences based on thymidine labeling [4], AML cell sorting into subpopulations followed by xeno-transplantation [5, 6], and *in-vitro* clonogenic assays [7], indicate that out of billions of AML blasts populating the bone marrow, only a minor fraction display sufficient self-renewal capacity to propagate the disease. Due to the similarity in the assays used to define self-renewing leukemic blasts and their functional resemblance to normal HSPC, these leukemic cells are designated leukemia stem cells (LSC) [8–11].

DNA-damaging agents in the form of cytarabine-anthracycline combination constitutes the mainstay of the remission induction therapy for the majority of AML subtypes for the last four decades [1]. Indeed, exponentially growing AML cells are rapidly killed by this genotoxic regimen and the majority of patients enter a remission stage. Unfortunately, AML cells grow back in more than 60% of the patients, causing leukemia relapse-thus, indicating LSC persistence during and after the treatment [3, 12]. It is therefore clear that these therapy-persistent cells represent the critical and largely unexplored target population in terms of therapy.

DNA double strand breaks generated via different modes of action by anti-leukemia drugs [13, 14], as well as by ionizing radiation (IR), initiate activation of intricate DNA damage response (DDR) signaling networks that alter cellular fate toward either survival or cell death. For some DDR factors, pro- (p53, PUMA) and anti-apoptosis (Bcl-2, Mcl-1) roles are well documented. In contrast, additional DDR genes (e.g. ATM, NF-kB, c-myc) may enhance chemosensitivity or confer resistance depending on the cellular context and drug type [15, 16]. In recent years the role of epigenetic modifiers in regulation of the DNA double strand break repair, cell cycle checkpoints and ultimately cell survival has emerged. Several lysine methyltransferases (KMTs), including G9a, Dot1L, SMYD2, EZH2 and Set7/9, were shown to regulate patterns of gene expression and cell fate via modifying key lysine residues on histones (H3, H4, H2B), transcription factors (p53, NF-kB), cell cycle regulators (Rb) and signaling kinases (MAPKAPK3) [17, 18]. As a result, small molecule inhibitors targeting some of these enzymes (e.g. DOT1L, EZH2) are currently in clinical trials for leukemia treatment [19].

Despite this remarkable progress it is clear that current DNA damaging and even targeted therapies unable to eliminate all leukemia regenerating cells, and thus, additional molecular determinants governing escape of these cells must exist and remain largely undefined. Given the high molecular and cellular heterogeneity of human AML and the growing appreciation of the complexity of the DDR, novel strategies that can pinpoint these resistance determinants should be developed in parallel.

Functional genomic screen, based on RNA interference mediated by shRNAs is a robust and unbiased approach to identify genes mediating resistance and sensitivity phenotypes [20, 21]. In this work we employed a whole genome shRNA screen to identify regulators of leukemia cell survival and regeneration after multiple rounds of genotoxic therapy. As a result we found that SMYD2 KMT knockdown confers relative resistance to multiple classes of DNA damaging agents. Induction of the transient dormancy in leukemia cells upon SMYD2 downregulation correlated with the increased DNA damage resistance, but make cells vulnerable to SET7/9 methyltransferase-specific inhibitor. AML patients with decreased SMYD2 have a lower likelihood of benefitting from standard chemotherapy. Thus, our study underscores the power of functional screening for resistance mediators and rationalizes SET7/9 pharmacological targeting in AML.

## RESULTS

### Genome wide shRNA screen identifies SMYD2 as a negative regulator of leukemia cell regeneration after genotoxic stress

**Figure 1:**
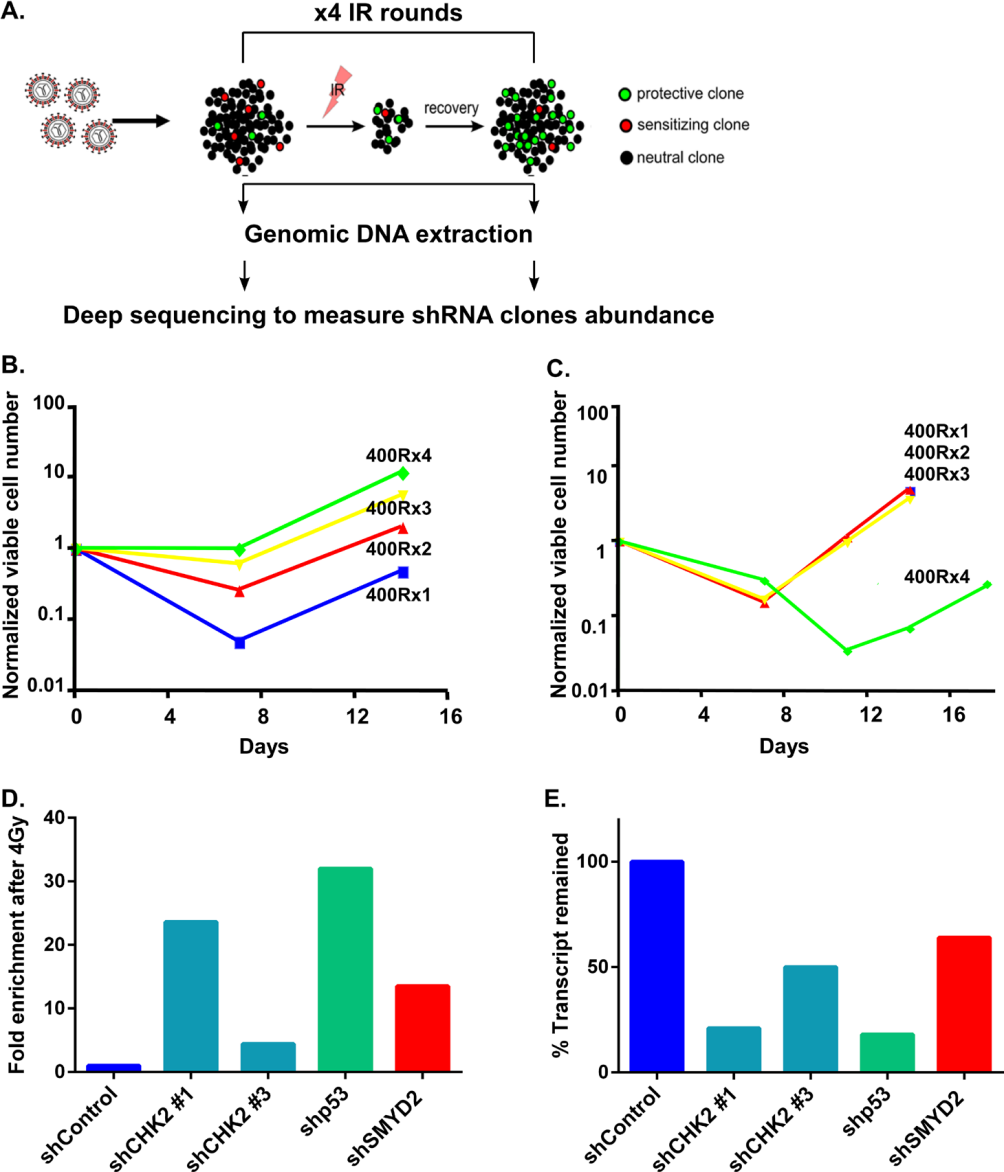
shRNA screen for regulators of leukemia cell survival (**A**) Experimental design of shRNA screen. TEX cells were infected with a genome scale lentivirus shRNAs library targeting 16,056 genes. Infected cells were irradiated with 4 Gy and left to recover. Recovered cells were treated with 3 more rounds of irradiation. After each round of irradiation DNA was extracted from recovered and non-treated cells followed by PCR amplification of the shRNA sequences. The composition of shRNA sequences was determined by deep sequencing. shRNA clonal dynamics in IR vs. non-treated cells schematically represented as enriched, depleted or unchanged with green, red and black colors respectively. (**B**) shRNA library infected TEX cells regeneration kinetics after sequential 4 rounds of IR with 4 Gy. (**C**) Parental TEX cells regeneration after sequential 4 rounds of IR with 4 Gy. (**D**) TEX cells infected with indicated shRNAs were mixed with TEX cells expressing shControl and EGFP. Then part of the culture was irradiated (4 Gy) or left untreated. Fold enrichment of EGFP- cells in IR vs. non-IR cultures was calculated on day 10 post IR. (**E**) QRT-PCR analysis of CHK2, p53 and SMYD2 genes following the respective shRNAs expression in TEX cells.

Regeneration of normal hematopoietic stem and progenitor (HSPC) as well as leukemia cells after DNA damage relies on cellular pathways that coordinate stress, survival and ultimately preservation of proliferative potential in the subset of viable cells [15]. IR potently suppresses normal HSPC regeneration via apoptosis and a number of cell death-independent pathways, including precipitous differentiation and senescence [22–25]. As such, numerous genes that participate in IR-induced DDR are key regulators of HSPC functions, including p53, ATM, Bcl2 and others [26]. To identify novel DDR regulators that mediate leukemia cell survival after IR, we utilized a pooled genome-wide lentiviral shRNA screen for genes that regulate cell recovery after 4 rounds of sublethal irradiation (4 Gy) (Figure 1A). In this screen we utilized the “stem cell-like” human hematopoietic cell line TEX generated via TLS-ERG leukemia fusion oncogene expression in cord-blood derived HSPC [27]. This leukemia model line maintains functional heterogeneity (only a minority of cells function as leukemia stem cells in a xenotransplantation setting), cytokine dependency (IL-3, SCF), and a functional p53 pathway. These features allowed us to use these cells as a “surrogate model” to study the biology of LSC [28]. A lentiviral shRNA library containing 80000 shRNA clones targeting approximately 16000 human genes was used to infect TEX cells at low multiplicity of infection to ensure representation of the library clones. Then one half of the library-transduced TEX cells were exposed to 4Gy of IR and the other half served as a control. This dose of IR (4Gy) resulted in the dramatic increase in cell death, such as only a low numbers of viable cells remained at 6–7 days post IR followed by the gradual regeneration (Supplementary Figure 1). We performed 4 cycles of IR and regeneration in order to enrich for the most potent shRNA clones and mimic multiple cycles of genotoxic therapy used in cancer treatment. Notably, TEX cells infected with the lentiviral shRNA library exhibited a remarkable increase in cell recovery rates following each round of sublethal irradiation, thus confirming the maintenance of regenerative potential and gain of enhanced survival (Figure 1B, compare 400RX1 with 400RX4). Non–infected, but similarly irradiated, parental cells did not demonstrate accelerated recovery, thereby ruling out the possibility of pre-existing resistant variants (Figure 1C, compare 400RX1 with 400RX4). We performed DNA deep sequencing of TEX cells regenerated after four rounds of IR and identified a set of shRNA clones strongly enriched in this experimental arm compared with the non-irradiated counterparts (Table 1) suggesting that the genes targeted by these shRNAs are important mediators of stress persistence and regeneration in leukemia cells. Indeed, known regulators of DDR such as CHK2 and p53 were identified in our screen (ranked 1 and 11 respectively from 15983 gene targets), thus validating our screen platform. In addition, a number of novel, potential regulators of leukemia cells DDR were found (Table 1). Validation experiment using TEX cells infected with individual shRNA targeting top candidates confirmed potent regeneration advantage of irradiated leukemia cells upon knockdown of CHK2, p53 and SMYD2 genes (Figure 1D). Enhanced regeneration was correlated with a reduction in the respective mRNA and protein levels pointing to the on-target effect (Figure 1E, Supplementary Figure 2). As the role of SMYD2 in leukemia cells growth and regeneration remains largely unexplored we decided to focus on this candidate.

**Table 1:** List of top candidates identified in the shRNA screen for leukemia cells survival and regeneration following genotoxic stress

Candidate rank*	Gene Symbol	Gene Name	IR/NT ratio of the most protective shRNA clone	most protective shRNA clone ID
1	CHEK2	Checkpoint Kinase 2, CHK2	3124	TRCN0000039946
2	NPY2R	neuropeptide Y receptor Y2	245	TRCN0000009214
3	CCL19	chemokine (C-C motif) ligand 19	8488	TRCN0000058014
4	SMYD2	SET and MYND domain containing 2	941	TRCN0000130403
5	UPF3B	UPF3 regulator of nonsense transcripts homolog B (yeast)	921	TRCN0000152769
6	CSPG4P5	chondroitin sulfate proteoglycan 4 pseudogene 5	3770	TRCN0000130902
7	FKBP10	FK506 Binding Protein 10	767	TRCN0000053931
8	UHMK1	U2AF homology motif (UHM) kinase 1	134	TRCN0000003281
9	CDNF	cerebral dopamine neurotrophic factor	59	TRCN0000133971
10	PAOX	polyamine oxidase (exo-N4-amino)	3.8	TRCN0000046254
11	TP53	tumor protein p53	35	TRCN0000003755

* Gene rank is calculated based on the representation of shRNAs before and after IR and reflects the potency of the shRNA clone to confer the phenotype after normalization for the differences in the shRNA pool size of various genes.

### SMYD2 knockdown rescues irradiated leukemia clonogenic cells in an apoptosis-independent manner

**Figure 2:**
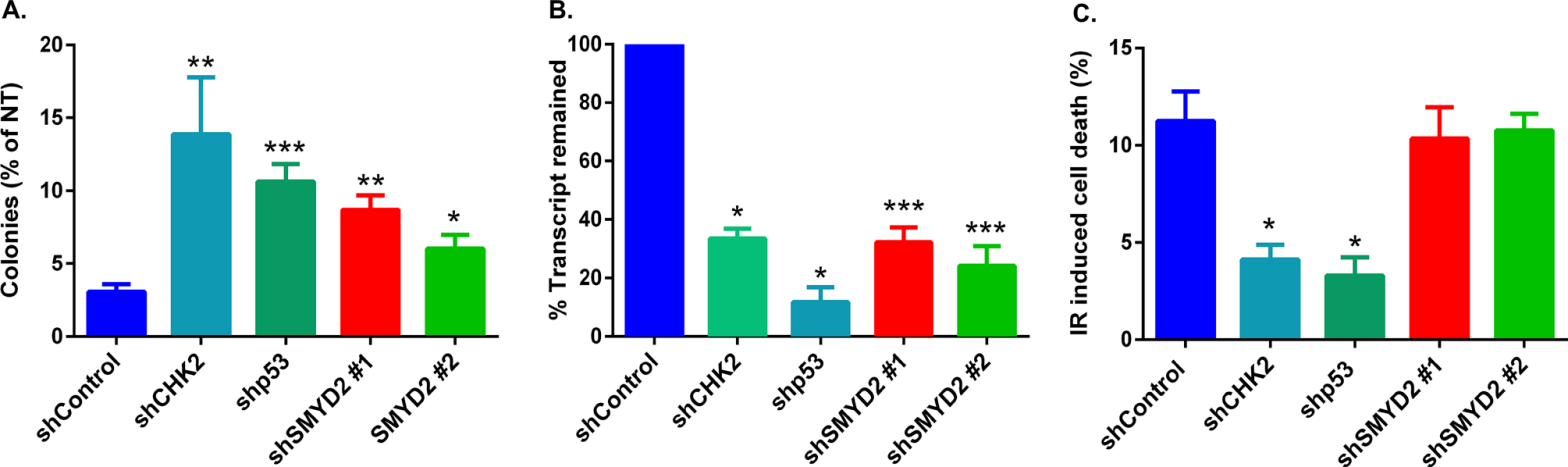
SMYD2 regulates leukemia cells response to genotoxic stress (**A**) Clonogenic potential of irradiated (4 Gy) OCI-AML2 cells expressing indicated shRNAs relative to the untreated counterparts. *n* = 3–8 independent experiments (*p < 0.05, **p < 0.005, ***p < 0.0001). (**B**) QRT-PCR analysis confirmed the reduction of mRNA levels of the examined genes (*p < 0.05, ***p < 0.0005). (**C**) Apoptosis was analyzed by Annexin V/Sytox staining and flow cytometry 18–24 hours after 4Gy IR. (*n* = 3–9, *p < 0.05). Bars represent means ± SEM.

To validate potential mechanisms by which SMYD2 participates in leukemia cells survival/recovery following DNA damage we measured IR-induced cell death upon SMYD2 knockdown. To that end we employed human leukemia line OCI-AML2 that possesses intact DNA damage regulators p53 [29] and CHK2 (this study) (Figure 2A). Irradiation (4Gy) of this line strongly diminished their clonogenic potential. SMYD2 knockdown using two independent SMYD2 shRNAs resulted in a 2-3 fold rescue of clonogenic capacity (Figure 2A). Similarly, SMYD2 knockdown in TEX cells led to the 4-fold increase in survival post Etoposide treatment as measured by the culture-initiating cell frequency analysis (Supplementary Figure 3). Knockdown of CHK2 and p53 genes also resulted in the rescue of irradiated OCI-AML2 clonogenic cells to a similar extent (Figure 2A). We confirmed the efficient knockdown of SMYD2, CHK2 and p53 genes with their respected shRNAs (Figure 2B). Decrease in the IR-induced apoptosis can be one of the mechanisms responsible for the rescue of irradiated cells clonogenic activity. To test this idea, we analyzed the rate of IR-induced cell death upon knockdown of SMYD2, p53 and CHK2 genes (Figure 2C). As expected, leukemia cells with p53 and CHK2 downregulation were less susceptible to IR-induced cell death in agreement with their role in this process. In contrast, downregulation of SMYD2 did not alter IR-induced cell death rate. These results suggest that the protective effect of SMYD2 on leukemia cell regeneration after irradiation is most probably apoptosis-independent.

### SMYD2 knockdown induces quiescence in leukemia cells

**Figure 3:**
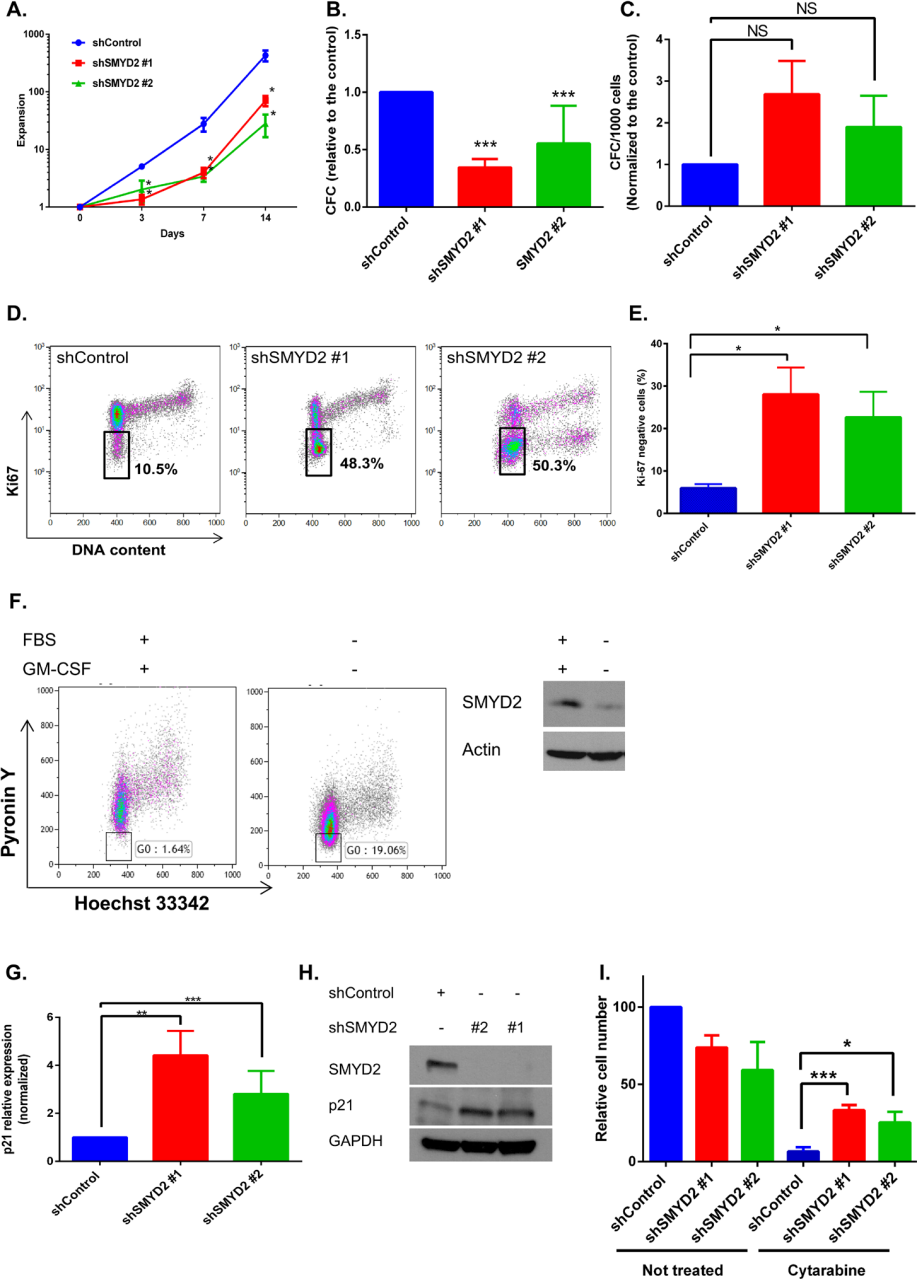
SMYD2 regulates leukemia cells growth and quiescence (**A**) OCI-AML2 cells were infected with indicated lentiviruses and selected with puromycin. Cells expansion was calculated by viable cell counting (*n* = 11, *p < 0.05). (**B**, **C**) Colony-forming ability of OCI-AML2 cells infected with the control or shSMYD2 viruses and plated into methylcellulose at D0 (*n* = 6–8, ***p < 0.0001) (B) or 13 days post infection (C). (**D**) Ki-67/DAPI representative flow cytometry analysis of OCI-AML2 cells infected with the control or shSMYD2 lentiviruses. Proportion of Ki-67 cells inside the gate is indicated. (**E**) Ki-67 negative cells analysis as in (D) from 3 independent experiments, *p < 0.05. (**F**) Hoechst 33342/PyroninY flow cytometry analysis of AML-193 cells upon FBS and GM-CSF starvation. Proportion of Pyronin Y_low_ cells (G0) inside the gate is indicated (left panel). Representative experiment is shown, *n* = 3. Western blot analysis of SMYD2 in actively proliferating and GM-CSF starved AML-193 cells (F, right panel), (*n* = 3). (**G**, **H**) Analysis of p21 mRNA (G) and protein (H) in OCI-AML2 expressing indicated shRNAs (*n* = 6–8, **p < 0.005, ***p < 0.0001). (**I**) Relative OCI-AML2 cell number expressing indicated shRNAs was measured using PrestoBlue. Cells were exposed to Cytarabine (1 μM) at the end of puromycin selection period (designated D0) (*n* = 4, *p < 0.05, ***p < 0.0005). Bars represent means ± SEM.

Ectopic expression of SMYD2 can either enhance [30, 31] or inhibit [32] cellular growth, probably acting in a cell type dependent manner. To investigate the role of the endogenous SMYD2 in leukemia cell proliferation we analyzed the expansion of OCI-AML2 cells infected with shSMYD2 lentiviruses. We detected a transient attenuation in cell growth rate without detectable changes in cellular viability upon SMYD2 knockdown that lasted for 5–7 days followed by the growth rate restoration (Figure 3A). In agreement with the described changes in proliferation, we observed an approximately 50% decrease in the frequency of clonogenic leukemia cells shortly after SMYD2 downregulation (3–5 days post infection) followed by a restoration of clonogenicity to the shControl levels at the later period (day 10 post infection) (Figure 3B, 3C). Of note, SMYD2 protein was consistently downregulated for the whole period of observation (Supplementary Figure 4). Taking into consideration the lower expansion rates of cells with diminished SMYD2 levels, we hypothesized that SMYD2 may affect cell cycle distribution. Using Ki-67 as a marker for cellular quiescence (G_0_), we revealed that up to 50% of OCI-AML2 cells with decreased levels of SMYD2 exist in the G_0_ state of the cell cycle at the end of puromycin selection period (designated D0) relative to only 10% of the control cells (Figure 3D, 3E). The fraction of Ki-67^-^ (quiescent cells) in shSMYD2 expressing cells gradually decreased to the shControl level by Day 7 in agreement with only transient attenuation of cell growth achieved by the constitutive SMYD2 knockdown (Supplementary Figure 5). To explore whether physiological regulators of leukemia cells quiescence, such as growth factors, can affect SMYD2 expression we utilized a growth-factor-dependent AML-193 leukemia line [33, 34]. In this model, GM-CSF withdrawal induces accumulation of cells in G_0_ as validated by the decreased Pyronin Y intensity (Figure 3F left panel). Western blot analysis revealed lower SMYD2 protein levels in the quiescence-enriched AML-193 cells relative to their actively dividing counterparts (Figure 3F right panel). These results reveal that leukemia growth factors can regulate SMYD2 levels. In correlation with the enlarged quiescent fraction upon SMYD2 knockdown we detected elevated levels of p21 mRNA and protein - observation consistent with its role in regulation of leukemia cells quiescence (Figure 3G, 3H) [35]. To provide a functional validation of the enhanced quiescence state induced upon SMYD2 knockdown we treated control and shSMYD2 expressing cells with a chemotherapeutic drug cytarabine that kills proliferating cells by interfering with DNA synthesis. Indeed, shSMYD2-expressing cells exhibited relative resistance to cytarabine (Figure 3I). Of note, whereas SMYD2 knockdown resulted in proliferation attenuation, SMYD2 overexpression did not affect OCI-AML2 cells proliferation or IR-induced cell death (Supplementary Figure 6). These results reveal that SMYD2 downregulation promotes the entry of leukemia cells into quiescence state associated with the increased resistance to anti-leukemia chemotherapy.

### SMYD2 downregulation induces p53-independent growth inhibition in leukemia cells

**Figure 4:**
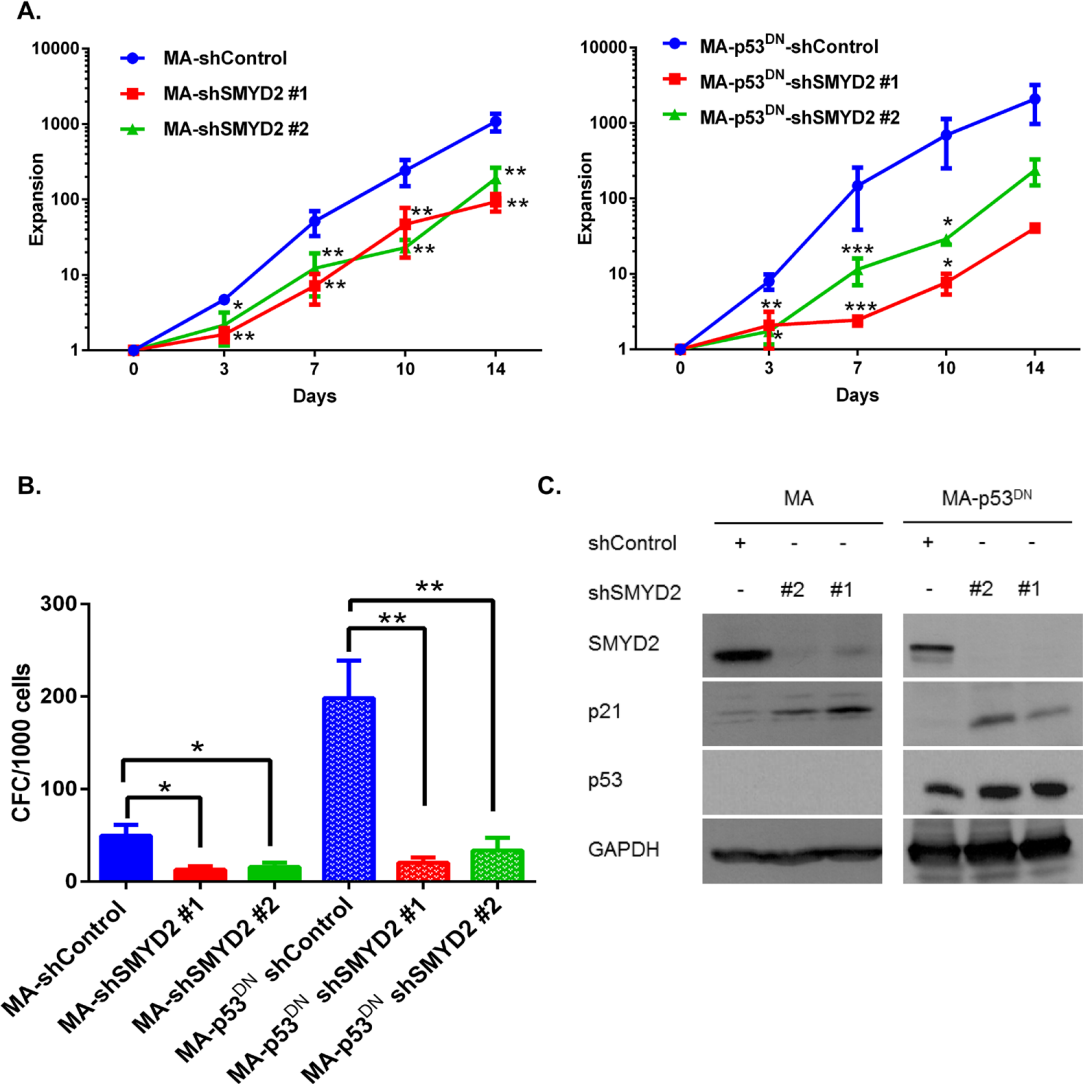
SMYD2 regulates leukemia cell growth in the p53-independent manner (**A**) Expansion of OCI-AML2 cells infected with the control (MA) or dominant negative p53 (MA-p53DN) and shSMYD2 lentiviruses was determined using viable counting (*n* = 5, *p < 0.05, **p < 0.005, ***p < 0.0005). (**B**) Clonogenic potential (CFC) of OCI-AML2 cells infected with the indicated lenti-constructs and plated in CFC assay at the end of puromycin selection period (designated D0). (*n* = 6, *p < 0.05, **p < 0.005). (**C**) Western blot analysis of p21, SMYD2 and p53 in OCI-AML2 cells infected with the indicated lentiviruses. Accumulation of inactive p53 protein is visible as a result of GSE56 overexpression. Representative image is shown, (*n* = 3). Bars represent means ± SEM.

Since p53 is one of the SMYD2 substrates [36] and a regulator of cell cycle progression [37], we inquired whether SMYD2 mediated leukemia cells growth arrest is mediated by p53. For that purpose we generated OCI-AML2 cells with the functionally inactive p53 via overexpressing GSE56 peptide that acts in a dominant-negative fashion (p53^DN^) and attenuates p53-mediated transactivation and apoptosis [22, 38]. SMYD2 downregulation resulted in the lower expansion rates of both control and p53^DN^ expressing cells (Figure 4A left and right panels). In agreement with the decreased proliferation rates, SMYD2 downregulation strongly reduced colony-forming ability of both control and p53^DN^ expressing cells (Figure 4B). Moreover, SMYD2 inactivation resulted in the measurable upregulation of p21 in the absence of the functional p53 (Figure 4C). These results demonstrate that SMYD2 downregulation can attenuate leukemia cell growth via p53-independent mechanisms.

### SMYD2 downregulation leads to SET7/9 upregulation in leukemia cells

**Figure 5:**
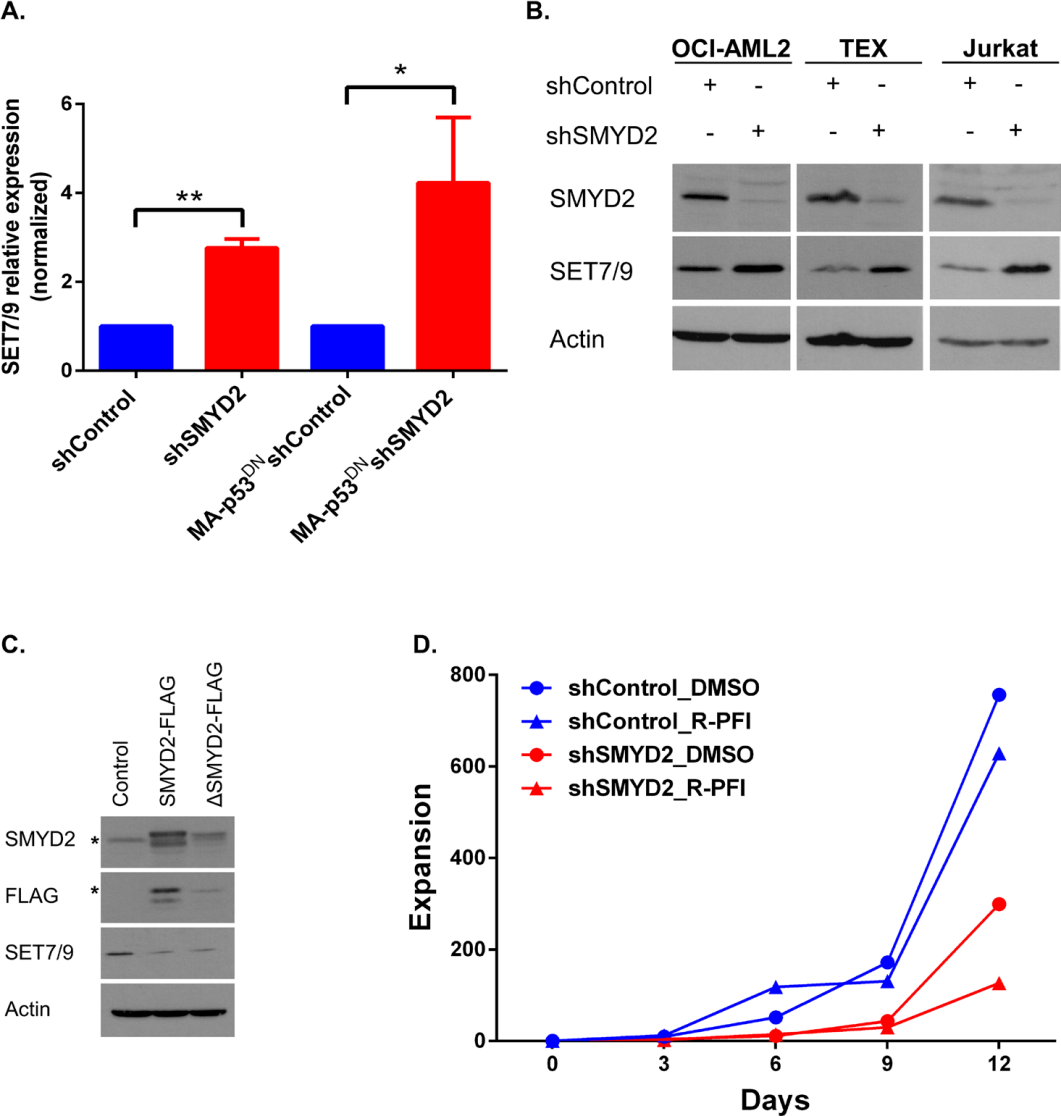
SMYD2 downregulation induces SET7/9 upregulation (**A**) Analysis of SET7/9 mRNA in the OCI-AML2 cultures (parental and p53^DN^) infected with shControl or shSMYD2 lentiviruses, (*n* = 4, *p < 0.05, **p < 0.0005). (**B**) Western blot analysis of SET7/9 and SMYD2 protein levels in OCI-AML2, TEX and Jurkat cells infected with control or shSMYD2 constructs. (**C**) Western blot analysis of SET7/9 levels upon ectopic expression of SMYD2-FLAG or SMYD2 ΔNHSC/ΔGEEV-FLAG dead enzyme expressing lentiviruses in OCI-AML2 cells. * indicates the specific band for the anti-FLAG or anti-SMYD2 antibodies. In each of the Western blots, actin levels were used as a loading control. (**D**) Expansion of OCI-AML2 cells expressing shControl or shSMYD2 and treated with a solvent (DMSO) or R-PFI2 (10 μM). Cells expansion was calculated by viable cells counting. Representative experiment out of three is shown. Complete data can be found in Supplementary Table 1.

Transient leukemia cell growth arrest upon SMYD2 downregulation followed by the return to the normal proliferation rate, as we reported above, can indicate activation of the compensatory mechanisms in response to changes in the SMYD2 levels and/or activity. In pursuance of a candidate gene that can overcome this SMYD2 knock-down mediated growth arrest we analyzed the expression of several KMTs with the similar structural and functional features to SMYD2 [39]. Our candidate methyltransferase list involved additional members of the SMYD family (SMYD3, SMYD4 and SMYD5), MLL family (MLL3 and MLL4) and structurally-related SET7/9 (SETD7). First, we examined their mRNA levels in OCI-AML2 and TEX cell lines infected with shSMYD2 and control shRNAs at the different time points post-infection (Supplementary Figure 7). Of those, only SET7/9 mRNA was consistently up regulated in the shSMYD2 expressing cells as compared to the control shRNA expressing cells (Figure 5A). Consistently with mRNA upregulation, SET7/9 protein levels were also increased upon SMYD2 knock-down in a number of cell lines tested (Figure 5B). We also revealed that SMYD2 ectopic expression led to the SET7/9 protein downregulation, further substantiating potential functional interactions between these two related KMTs (Figure 5C). In order to test whether SET7/9 upregulation in cells with diminished SMYD2 levels is functionally important we utilized recently described inhibitor of SET7/9 methyltransferase activity (R)-PFI-2 [40]. We found that (R)-PFI-2 inhibited the growth of OCI-AML2 cells expressing shSMYD2, whereas proliferation of the shControl expressing cells was mostly unaffected (Figure 5D and Supplementary Table 1). Collectively, these results reveal the existence of the functional cross-talk between SMYD2 and SET7/9 lysine methyltransferases.

### Decreased SMYD2 expression correlates with poor therapy response in primary AML

**Figure 6:**
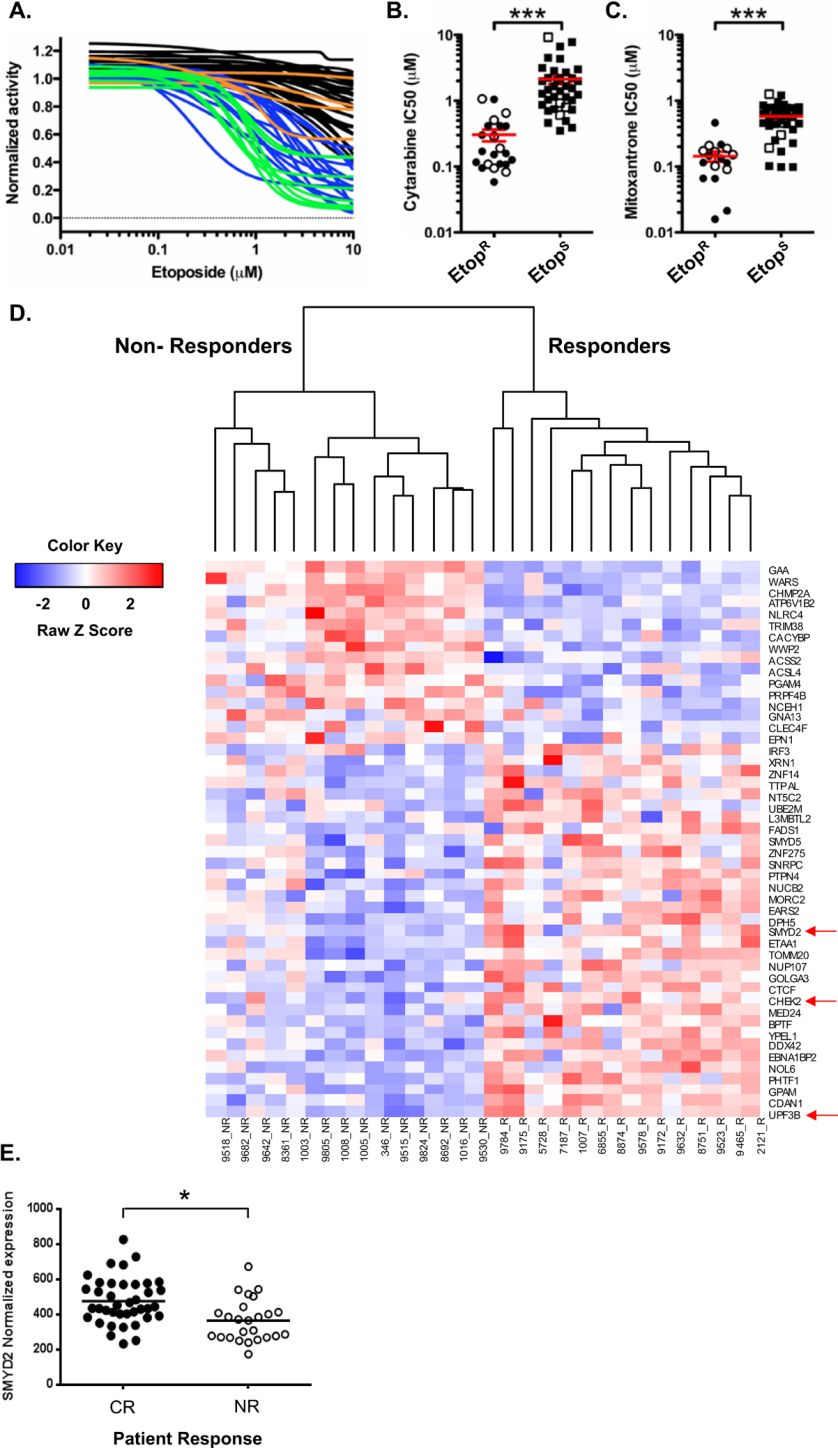
High SMYD2 expression predicts AML response to therapy (**A**) Sensitivity to Etoposide of primary AML and CML samples. Primary AML and CML cells were treated with Etoposide and growth was measured by PrestoBlue Reagent. “Etoposide resistance (EtopR)” was determined by curve analysis and inhibition at 10 μM. AML EtopS (sensitive)–blue; AML EtopR–black; CML EtopS–green; CML EtopR–orange. IC50 analysis of primary AML (closed symbols) and CML (open symbols) cells treated with (**B**) cytarabine and (**C**) mitoxantrone. In A-C panels, relative cell growth was quantitated using PrestoBlue Cell Viability Assay. (Wilcoxon-rank sum test* *p* < 0.05; ***p < 0.001). (**D**) Illumina beadchip array based expression analysis of the shRNA hits that are differentially expressed in Etoposide responders versus non-responders showing. SMYD2, CHEK2 and UPF3B genes are among the top 11 shRNA hits (Table 1) and indicated by the red arrow. High and low expression is indicated by red and blue colors respectively, (FDR ≤ 0.05). (**E**) SMYD2 expression level analysis in AML patients that achieved complete response (CR) or non-response (NR), *p* < 0.05.

Our results using cell lines revealed the involvement of SMYD2 in regulation of leukemia cell growth, survival and regeneration after genotoxic stress. To evaluate whether changes in SMYD2 levels are significant factor in determining primary AML drug sensitivity and patient survival we utilized leukemia patient derived samples. Firstly, we determined the sensitivity of a wide spectrum of primary AML and CML samples (*N* = 63) to the DNA damaging drug Etoposide (Figure 6A). Primary samples were incubated with Etoposide for 24 hrs followed by the 72 hrs long recovery period prior to viability determination using Presto blue based colorimetric assay. We found that primary leukemia samples exhibited heterogeneous sensitivity to Etoposide, which allowed separation into two groups. Those AML samples whose growth was inhibited at 10 μM Etoposide were designated “Etoposide sensitive, (Etop^S^)”, whereas the less affected samples constituted the “Etoposide resistant, (Etop^R^)” group. Importantly, Etop^S^ samples also exhibited higher susceptibility to the additional genotoxic agents Cytarabine and Mitoxantrone relative to the Etop^R^ group (Figure 6B and 6C). Of note, these chemotherapeutics are standard of care in AML treatment. Consistency in sensitivity profiles across multiple drugs validated this AML samples set as a useful platform to investigate molecular regulators responsible for the differential therapy sensitivity [28 and unpublished]. Gene expression profiling of Etop^S^ (*n* = 12) and Etop^R^ (*n* = 12) samples was undertaken to characterize gene signatures associated with chemotherapy response. To assess whether genes identified in our functional shRNA screen (Table 1, top 200 candidates) play a role in Etoposide sensitivity determination of primary AML samples, we analyzed the overlap between the two datasets. This analysis revealed 49 candidates (out of the top 200) that were also differentially expressed (FDR < 0.05) in Etop^S^ and Etop^R^ samples (Figure 6D). More significantly, 33 out of 49 candidates, including functionally validated SMYD2 and CHK2 genes, demonstrated significantly lower expression in Etop^R^ samples (Figure 6D). This association is in agreement with the overall functional screen rationale and strongly supports our results demonstrating that SMYD2 knockdown confers relative resistance to genotoxic stress.

To test whether SMYD2 levels can predict the response of AML patients to chemotherapy we measured its mRNA abundance in peripheral blood or bone marrow blasts of 71 AML patients treated according to a standard remission induction protocol (Cytarabine and Daunorubicin combination) [41]. The average SMYD2 expression level in 39 patients that achieved complete remission (CR) was significantly higher than that in 25 patients that had no-response (NR) (*p* < 0.05) (Figure 6E). These results indicate that AML patients with elevated SMYD2 have a greater likelihood of benefitting from standard chemotherapy. Of interest, extracting SMYD2 expression values from the Oncomine cancer array database [42] revealed that SMYD2 levels in AML are lower than in Acute Lymphoblastic Leukemia (ALL) (Supplementary Figure 8).

Collectively, our analysis of primary AML samples revealed that higher SMYD2 expression correlates with the increased susceptibility to genotoxic chemotherapy and favorable outcome.

## DISCUSSION

Molecular determinants of AML resistance to standard DNA damaging agents remain elusive and account, at least partially, for the treatment failure. Expression profiling and more recently high throughput DNA sequencing technologies provided biomedical community with rich inventory of genes that are aberrantly expressed or function in AML. These powerful descriptive methods also highlighted high genetic and epigenetic heterogeneity of human AML [43–45]. However, the genes that actually mediate therapy resistance and thus directly impact the treatment outcome and patients’ survival is difficult to extract from these datasets. In this study we utilized a genome-wide functional screen to rapidly enrich for shRNAs that confer relative regenerative advantage to leukemia cells after repetitive DNA damage insults. In addition to SMYD2, whose role in AML sensitivity to DNA damage is novel and discussed below, our top list shRNAs included several well-annotated (p53 and CHK2) and proposed (PAOX [46]) determinants of cellular resistance to stress. Beyond these genes, our functional screen also identified numerous radiation resistance conferring shRNA clones that target genes previously unconnected with DNA damage response and that await experimental validation. These positive results further highlight the power of functional genetic [20, 47] and small molecules based [28, 48] screens to identify novel resistance determinants.

Cell survival at the onset of genotoxic injury relies on the activation of the signaling pathways regulating DNA repair, cell cycle checkpoints and cell death [15]. In response to IR, cells in which SMYD2 was downregulated underwent cell death at the levels similar to the parental control line. Despite the similarity in the cell death rates, irradiated shSMYD2 expressing cells formed more colonies in a clonogenic assay pointing to the involvement of cell death independent mechanisms. Indeed, careful examination of the cell cycle distribution revealed strongly elevated quiescent (G_0_) and reduced S phase compartments in leukemia cells with decreased SMYD2 levels. This transient quiescence is likely contributes to the observed radio- and cytarabine resistance in our experiments and was postulated to be a critical mechanism for therapy resistance of normal and leukemic stem cells in patients and mouse models [49]. The involvement of SMYD2 in regulation of cell proliferation was suggested previously. Indeed, overexpression of SMYD2 stimulated proliferation of esophageal squamous cancer cell lines, but inhibited growth of murine fibroblasts and Embryonic Stem Cells indicating strong cell type dependency [31, 32, 50]. Moreover, knockdown of SMYD2 attenuated growth of several epithelial and mesenchyme derived cancer lines [30, 31]. SMYD2 mediated methylation with subsequent changes in the activity of a plethora of cellular proteins can account for its regulation of cell cycle, quiescence and survival. Indeed, SMYD2 methylation of histone H3 on lysine 36 and lysine 4 reported to promote repression [32] and activation [51] of the distinct gene subsets. In addition, SMYD2-dependent methylation alters the activity of nuclear and cytoplasmic non-histone proteins including p53 [36], Rb [30, 52], estrogen receptor alpha [53], Hsp90 [54, 55], PTEN [56], MAPKAPK3 [57] and numerous other [58–60]. Transcriptional induction of p21 cell cycle inhibitor in leukemia cells with decreased SMYD2 levels can also account for the observed transient quiescence [61] and more efficient DNA damage response critical for the enhanced survival [35].

p53 transcription factor regulates p21 expression [62], promotes cell cycle arrest [37, 63] and also undergoes SMYD2-dependent repression via methylation on Lys370 [36]. Thus, p53/p21 axis can account for SMYD2 mediated growth arrest. In the leukemia cell lines model we employed, SMYD2 downregulation induced p21 and cell growth inhibition in a p53-independent manner. Similarly, SMYD2 knockdown reduced proliferation of cells expressing mutant or no p53 [31]. Although the exact mechanism/s by which SMYD2 deregulation leads to p21 induction remains to be identified several mechanisms can be proposed. For instance, knockdown of SMYD2 inhibited Akt phosphorylation via decreased PTEN methylation that resulted in the growth suppression of breast cancer cells [56]. Under these circumstances, FoxO transcription factor will undergo activation and can induce p21 expression [64]. Recent study also implicated SET7/9 in the regulation of E2F1 transcriptional targets, including p73, that is known regulator of p21 [65, 66]. Given these complexity, multiple rather than a single SMYD2 target might be implicated in its effect on leukemia cell growth.

Upregulation of SET7/9 KMT mRNA and protein specifically in SMYD2 knockdown cells, as we discovered in this study, can provide a clue toward the cellular substrate and molecular pathways involved in SMYD2-mediated growth regulation and stress resistance. A noticeable feature of the methyltransferase-substrate network in general, and of SET7/9 and SMYD2 KMTs in particular, is their substrate promiscuity so that different KMTs can methylate the same protein regulating its activity [18, 67]. Indeed, SMYD2 and SET7/9 bind and modify the activity of histone H3, p53, Rb, ERa and possibly other proteins. Smyd2-dependent binding, methylation and regulation of SET7/9 promoter associated transcription factors might provide plausible mechanism for the discovered interplay between the two KMTs. Of note, SET7/9 and SMYD2 methylation on p53, for example, are mutually inhibitory and the crosstalk between them and additional posttranslational modifications can lead to the distinct functional outcomes under different biological conditions [18, 67, 68]. Enhanced sensitivity of SMYD2 knockdown cells to the specific SET7/9 inhibitor supports the notion that these two KMTs converge on the regulation of the same substrate and/or pathway.

In this study we revealed that SMYD2 under-expression correlated positively with the resistance of primary human AML samples to several genotoxic agents. Importantly, we have revealed that low SMYD2 expression predicted no-response of the AML patients to the standard induction chemotherapy. In line with our findings in AML, low levels of SMYD2 correlated with shorter survival in patients with renal cell carcinoma [69] and acquisition of complex karyotype and disease progression in chronic lymphocytic leukemia [70]. In other tumor types, including esophageal squamous cell carcinoma [31], gastric carcinoma [71], acute lymphoblastic leukemia (ALL) [72] and HPV-unrelated head and neck carcinoma [73], SMYD2 overexpression was associated with disease aggressiveness and worse outcome. These seemingly opposing clinical observations might stem from the tissue-specific or disease stage-specific functions of SMYD2. For instance, many fold higher expression of SMYD2 in ALL vs. AML, as revealed by our bioinformatics analysis might be required to sustain the rapid proliferation of ALL blast cells [74]. In contrast to ALL, low levels of SMYD2 can contribute to the maintenance of the largely quiescent AML stem cells [75]. Future study of SMYD2 function in the functionally different AML cellular compartments might validate this hypothesis. Genetically engineered murine models of cancer further emphasize tissue- specific and oncogene-specific roles of SMYD2 in tumorigenesis. Indeed, complete ablation of SMYD2 in the K-Ras/p53^-/-^ model of pancreatic adenocarcinoma [57] and MLL/AF9-N-Ras leukemia model [76] revealed minor delay in the disease onset or no difference in Myc-induced lymphomagenesis [76].

In conclusion, we revealed that SMYD2 levels regulate leukemia cell proliferation and response to genotoxic stress. We propose that the interplay between SMYD2 and SET7/9 KMTs levels shifts leukemia cells from growth to quiescence state that is associated with the higher resistance to several DNA damaging agents. Better understanding of the methylation targets and molecular pathways affected by these enzymes might be beneficial for the rationale use of the small molecule KMT inhibitors in AML and other types of cancer.

## MATERIALS AND METHODS

### Cell culture and reagents

TEX hematopoietic cell line was described elsewhere [27]. TEX cells were cultured in Iscove's Modified Dulbecco's Medium (IMDM, Gibco, Israel) supplemented with Fetal Bovine Serum (FBS, 15%, MultiCell, Canada), SCF (20ng/ml), IL-3 (2ng/ml), L-Glutamine (L-Glu,1%) and Penicillin/Streptomycin (P/S, 1%). OCI-AML2 leukemia cell line was cultured in RPMI medium supplemented with FBS (10%, Biological Industries, Israel), L-Glu (1%) and P/S (1%). AML-193, a cytokine-dependent human leukemia cell line was purchased from ATCC (CRL958) and cultured in IMDM supplemented with BIT-9500 (5%, Stem Cell Technologies, Canada), FBS (5%), GM-CSF (5ng/ml), L-Glu (1%) and P/S (1%). Jurkat leukemia cells were cultured in RPMI supplemented with FBS (20%), L-Glu (1%) and P/S (1%). Primary AML and chronic myelogenous leukemia (CML) cells were cultured in IMDM, BIT-9500 (10%), low-density lipoproteins (5 mg/mL), 2-mercaptoethanol (55 μM), L-Glu (1%), P/S(1%), SCF (100 ng/mL), FLT3-ligand (100 ng/mL), G-CSF (20 ng/mL), IL6 (20 ng/mL), TPO (50 ng/mL), IL-3 (20 ng/mL), GM-CSF (20 ng/mL). All cytokines were from Peprotec, Asia. All cells were maintained in a humidified incubator at 37°C and 5% CO_2_. All cell lines were tested negative for mycoplasma.

### Clinical samples

Primary human AML and chronic myelogenous leukemia (CML) cells were isolated from peripheral blood according to procedures approved by the University Health Network Research Ethics Board (Toronto, ON) and their response to Etoposide was determined as described in drug sensitivity analysis. AML samples with known clinical response to induction chemotherapy (complete remission vs. non responders) were described elsewhere [41].

### Viral constructs

Lentiviral construct expressing shSMYD2 and shGFP587 (shControl) were cloned into pLKO-puro plasmids. Lentiviral construct expressing dominant negative p53 (MA1-GSE56) was described previously [22]. The following TRC lentiviral shRNA clones were used in validation study: shSMYD2 (TRCN0000130403, TRCN0000130774), shp53 (TRCN0000003755), shCHK2 (TRCN0000039946), shControl (TRCN0000231746). For overexpression experiments, SMYD2-FLAGX3 and SMYD2-ΔNHSC/ΔGEEV-FLAG dead enzyme were cloned by NsiI and SphI into pMin-SFFV viral vector. pLVTHM plasmid served as control.

### Virus preparation and transduction procedure

4 × 10^6^ 293T cells were transfected with CMVdeltaR8 (2.5 μg), pMDG (0.28 μg) and pLKO (2.8 μg) using PolyJet transfection reagent (SignaGen Laboratories, BioConsult, Israel) according to the manufacturer's instructions. After 24 h, the medium was replaced with DMEM supplemented with 10% FBS, 1% P/S and 1% L-Glu. The following day the supernatants were centrifuged for 2 h in 22,000 rpm, 4*°C*. The concentrated viruses were resuspended in IMDM supplemented with 1%BSA. Leukemia cells were infected by addition of viral supernatant (MOI 0.5) and polybrene (4 μg/ml) to the cells followed by centrifugation for 30 min, 1500 rpm and allowed to recover for 24 h. Next day cells were washed from the virus and incubated for additional 24 hours followed by Puromycin addition. The following selection conditions were used: Jurkat (0.3 μg/ml Puromycin for 3 days), OCI-AML2 (2 μg/ml Puromycin for 3 days). After selection completion, cells were replated for the downstream experiments. This point was designated “day 0”.

### Drug sensitivity, cell cycle and viability analysis

Etoposide and Ara-C (both from Sigma, Israel) were added to cells at the indicated concentrations for 24 h followed by drug wash. Cells were cultured in drug-free medium for 72 hours and relative cell growth was quantitated using PrestoBlue^®^ Cell Viability Assay (ThermoFisher Scientific, Rhenium, Israel) using a multiplate reader (SpectraMax 340PC, Molecular Devices Corp, USA) according to manufacturer's protocols. Cells were exposed to ionizing radiation using Cs-137 source at the dose rate 2.8 Gy/min using GMBH BioBeam 8000 gamma irradiation device (Gamma service, UK).

To quantitate clonogenic growth potential of the leukemic cells we plated shControl and shSMYD2 expressing OCI-AML2 cells (500 cells/plate and 5000 cells/plate respectively) in MethoCult^™^ H4100 (Stem Cell Technologies, Canada) supplemented with FBS (30%, MultiCell, Canada), 5637 cells conditioned medium (10%), L-Glu (1%) and P/S (1%), 2-mercaptoethanol (50 μM). Cells were plated at day 0 or at day 10 from the end of puromycin selection. Colonies were counted under the microscope after 14 days of incubation.

To induce quiescence, AML-193 leukemia cells were washed three times with PBS and replated into IMDM supplemented with BIT-9500 for 4 days. Quiescent fraction was determined using Hoechst 33342 /Pyronin Y staining. Briefly, 1 × 10^6^ cells were stained with Hoechst 33342 (10 μg/ml, Life Technologies, Rhenium, Israel), incubated for 45 min at 37°C followed by addition of 500 ng/ml Pyronin Y (Sigma, Israel) for additional 15 min.

For Ki-67 analysis, cells transduced with different shRNAs were fixed in formaldehyde (1.4%), permeabilized in ethanol (100%) followed by labeling with Ki-67 antibody conjugated with AxFluor488 (Invitrogen). DAPI (4′,6-diamidino-2-phenylindole, 1 μg/ml) was added to visualize DNA content.

Apoptosis was measured using Annexin-V and SYTOX^™^ Blue *dead cell stain* (Invitrogen) according to manufacturer's protocols. Cells were analyzed with Gallios flow cytometer (Beckman Coulter). Flow cytometry data were analyzed using Kaluza flow cytometry analysis software (Beckman-Coulter).

Inhibition of SET7/9 was done by adding DMSO or 10 μM of (R)-PFI2 to 1 × 10^5^ OCI-AML2 cells infected with shControl or shSMYD2. Every 3 days cells were counted and 1 × 10^5^ cells were replated with fresh DMSO or SET7/9 inhibitor. Cell expansion was calculated by viable cell counting using Trypan Blue dye.

### Genome-wide shRNA library screen

Logarithmically growing TEX cells (80 × 10^6^ cells) were infected with a genome scale lentivirus library consisting of ∼78,432 shRNAs targeting 16,056 unique RefSeq genes [77]. Cells were infected at multiplicity of infection that results in 30% survival after puromycin selection (0.7 μg/ml for 48 hours) to ensure a single shRNA clone integration per cell. After puromycin selection completion, dead cells were removed by washing and the culture was divided for irradiation (4Gy) and control experimental arms (30 or 60 × 10^6^ cells respectively). The reference sample (D0, 30 × 10^6^) was frozen down. After irradiation, cells were allowed to regenerate with regular medium changes till their number reached that of the initial input (∼14 days). At this point (designated 4 Gy × 1) part of the cells were exposed to the second dose of 4Gy and left for an additional regeneration cycle (4 Gy × 2). In total, for this screen TEX cells were exposed to 16Gy of IR. Aliquots (30 × 10^6^ cells) of irradiated and sham treated cells were collected at different time points. Genomic DNA was extracted using the QIAamp Blood Maxi Kit (Mississauga, ON, Canada) according to the manufacturer's recommendation. Genomic DNA was extracted from triplicate D0, D10, D21, D31 non-irradiated and 4 Gy × 1, 4 Gy × 2, 4 Gy × 3, 4 Gy × 4 survivors. pLKO1 vector derived primers flanking unique shRNA sequences were used for PCR amplification to generate a library of shRNA products for next generation sequencing. From sequencing reads, the abundance of shRNA clone in the non-treated and irradiated samples was determined. shRNA clones that demonstrated relative enrichment in the repeatedly irradiated samples were selected for further validation.

### Quantitative RT-PCR

RNA was extracted with the Trizol reagent (Invitrogen) and reverse transcribed with SuperScript III (Invitrogen). Real-time PCR reactions were prepared with SYBR Green PCR Master Mix (Applied Biosystems) in triplicates and analyzed on Applied Biosystems 7900HT instruments. Absolute gene expression was quantified with SDS software (Applied Biosystems) based on the standard curve method and presented after normalization for GAPDH.

Real Time PCR primers: SMYD2, Fwd. AATCCACCCAGAGAGAACAC, Rev. AGTGATGGAG AGCAGCTATG;GAPDH, Fwd. ACCCACTCCTCCACC TTTGA, Rev. CTGTTGCTGTAGCCAAATTCGT;p53, Fwd. CCCAAGCAATGGATGATT TGA, Rev. GGCATTC TGGGAGCTTCATCT;CHK2, Fwd. TCGAAAGCCAGC TTTACCTC, Rev. TGATCAGTCAGTTTATCCTAAGGC; p21, Fwd. CGCGACTGTGATGCGCTAATG, Rev. GGAA CCTCTCATTCAACCGCC; SET7/9, Fwd. TTGAGGGG AACTTTGTTCA, Rev. CAGCTCTCCGTCTACATACG.

### Western blotting analysis

The following primary antibodies were used: mouse anti-β-actin (clone 8H10D10, Cell Signaling, 1:2000), Goat anti human SMYD2 (c-20, Santa Cruz, 1:500), rabbit anti human p21 (c-19, Santa Cruz, 1:400), Mouse anti-Flag (Santa Cruz, 1:500), Mouse anti-p53 (Invitrogen, PAB1801, 1:1000), anti-CHEK2 phospho-Thr68 (clone C13C1, Cell Signaling, 1:2000).

### Statistical analysis

The significance of differences among groups was determined by Student's *t* test test (Excel software). Wilcoxon-rank sum test was used in Figure 6.

## SUPPLEMENTARY FIGURES AND TABLE



## References

[1] Dohner H, Weisdorf DJ, Bloomfield CD (2015). Acute Myeloid Leukemia. N Engl J Med.

[2] Greenlee RT, Hill-Harmon MB, Murray T, Thun M (2001). Cancer statistics, 2001. CA Cancer J Clin.

[3] Deschler B, Lubbert M (2006). Acute myeloid leukemia: epidemiology and etiology. Cancer.

[4] Gavosto F, Pileri A, Bachi C, Pegoraro L (1964). Proliferation and Maturation Defect in Acute Leukaemia Cells. Nature.

[5] Lapidot T, Sirard C, Vormoor J, Murdoch B, Hoang T, Caceres-Cortes J, Minden M, Paterson B, Caligiuri MA, Dick JE (1994). A cell initiating human acute myeloid leukaemia after transplantation into SCID mice. Nature.

[6] Bonnet D, Dick JE (1997). Human acute myeloid leukemia is organized as a hierarchy that originates from a primitive hematopoietic cell. Nat Med.

[7] Buick RN, Chang LJ, Messner HA, Curtis JE, McCulloch EA (1981). Self-renewal capacity of leukemic blast progenitor cells. Cancer Res.

[8] Dick JE (2008). Stem cell concepts renew cancer research. Blood.

[9] Valent P, Bonnet D, De Maria R, Lapidot T, Copland M, Melo JV, Chomienne C, Ishikawa F, Schuringa JJ, Stassi G, Huntly B, Herrmann H, Soulier J (2012). Cancer stem cell definitions and terminology: the devil is in the details. Nat Rev Cancer.

[10] Pei S, Jordan CT (2012). How close are we to targeting the leukemia stem cell?. Best practice & research Clinical haematology.

[11] Stubbs MC, Armstrong SA (2007). Therapeutic implications of leukemia stem cell development. Clin Cancer Res.

[12] Jordan CT (2005). Targeting the most critical cells: approaching leukemia therapy as a problem in stem cell biology. Nat Clin Pract Oncol.

[13] Kurz EU, Douglas P, Lees-Miller SP (2004). Doxorubicin activates ATM-dependent phosphorylation of multiple downstream targets in part through the generation of reactive oxygen species. J Biol Chem.

[14] Grant S (1998). Ara-C: cellular and molecular pharmacology. Advances in cancer research.

[15] Roos WP, Thomas AD, Kaina B (2016). DNA damage and the balance between survival and death in cancer biology. Nat Rev Cancer.

[16] Galluzzi L, Vitale I, Vacchelli E, Kroemer G (2011). Cell death signaling and anticancer therapy. Frontiers in oncology.

[17] Chen Y, Zhu WG (2016). Biological function and regulation of histone and non-histone lysine methylation in response to DNA damage. Acta biochimica et biophysica Sinica.

[18] Biggar KK, Li SS (2015). Non-histone protein methylation as a regulator of cellular signalling and function. Nat Rev Mol Cell Biol.

[19] Wagner T, Jung M (2012). New lysine methyltransferase drug targets in cancer. Nat Biotechnol.

[20] Porter CC, Kim J, Fosmire S, Gearheart CM, van Linden A, Baturin D, Zaberezhnyy V, Patel PR, Gao D, Tan AC, Degregori J (2012). Integrated genomic analyses identify WEE1 as a critical mediator of cell fate and a novel therapeutic target in acute myeloid leukemia. Leukemia.

[21] Sullivan KD, Padilla-Just N, Henry RE, Porter CC, Kim J, Tentler JJ, Eckhardt SG, Tan AC, Degregori J, Espinosa JM. ATM (2012). MET kinases are synthetic lethal with nongenotoxic activation of p53. Nat Chem Biol.

[22] Milyavsky M, Gan OI, Trottier M, Komosa M, Tabach O, Notta F, Lechman E, Hermans KG, Eppert K, Konovalova Z, Ornatsky O, Domany E, Meyn MS (2010). A distinctive DNA damage response in human hematopoietic stem cells reveals an apoptosis-independent role for p53 in self-renewal. Cell Stem Cell.

[23] Meng A, Wang Y, Van Zant G, Zhou D (2003). Ionizing radiation and busulfan induce premature senescence in murine bone marrow hematopoietic cells. Cancer Res.

[24] Wang J, Morita Y, Han B, Niemann S, Loffler B, Rudolph KL (2016). Per2 induction limits lymphoid-biased haematopoietic stem cells and lymphopoiesis in the context of DNA damage and ageing. Nat Cell Biol.

[25] Wang J, Sun Q, Morita Y, Jiang H, Gross A, Lechel A, Hildner K, Guachalla LM, Gompf A, Hartmann D, Schambach A, Wuestefeld T, Dauch D (2012). A differentiation checkpoint limits hematopoietic stem cell self-renewal in response to DNA damage. Cell.

[26] Bakker ST, Passegue E (2013). Resilient and resourceful: genome maintenance strategies in hematopoietic stem cells. Exp Hematol.

[27] Warner JK, Wang JC, Takenaka K, Doulatov S, McKenzie JL, Harrington L, Dick JE (2005). Direct evidence for cooperating genetic events in the leukemic transformation of normal human hematopoietic cells. Leukemia.

[28] McDermott SP, Eppert K, Notta F, Isaac M, Datti A, Al-Awar R, Wrana J, Minden MD, Dick JE (2012). A small molecule screening strategy with validation on human leukemia stem cells uncovers the therapeutic efficacy of kinetin riboside. Blood.

[29] Yang GS, Minden MD, McCulloch EA (1993). Influence of schedule on regulated sensitivity of AML blasts to cytosine arabinoside. Leukemia.

[30] Cho HS, Hayami S, Toyokawa G, Maejima K, Yamane Y, Suzuki T, Dohmae N, Kogure M, Kang D, Neal DE, Ponder BA, Yamaue H, Nakamura Y (2012). RB1 methylation by SMYD2 enhances cell cycle progression through an increase of RB1 phosphorylation. Neoplasia.

[31] Komatsu S, Imoto I, Tsuda H, Kozaki KI, Muramatsu T, Shimada Y, Aiko S, Yoshizumi Y, Ichikawa D, Otsuji E, Inazawa J (2009). Overexpression of SMYD2 relates to tumor cell proliferation and malignant outcome of esophageal squamous cell carcinoma. Carcinogenesis.

[32] Brown MA, Sims RJ, Gottlieb PD, Tucker PW (2006). Identification and characterization of Smyd2: a split SET/MYND domain-containing histone H3 lysine 36-specific methyltransferase that interacts with the Sin3 histone deacetylase complex. Molecular cancer.

[33] Lange B, Valtieri M, Santoli D, Caracciolo D, Mavilio F, Gemperlein I, Griffin C, Emanuel B, Finan J, Nowell P (1987). Growth factor requirements of childhood acute leukemia: establishment of GM-CSF-dependent cell lines. Blood.

[34] Jedema I, Barge RM, Frankel AE, Willemze R, Falkenburg JH (2004). Acute myeloid leukemia cells in G0 phase of the cell cycle that are unresponsive to conventional chemotherapy are sensitive to treatment with granulocyte-macrophage colony-stimulating factor/diphtheria toxin fusion proteins. Exp Hematol.

[35] Viale A, De Franco F, Orleth A, Cambiaghi V, Giuliani V, Bossi D, Ronchini C, Ronzoni S, Muradore I, Monestiroli S, Gobbi A, Alcalay M, Minucci S (2009). Cell-cycle restriction limits DNA damage and maintains self-renewal of leukaemia stem cells. Nature.

[36] Huang J, Perez-Burgos L, Placek BJ, Sengupta R, Richter M, Dorsey JA, Kubicek S, Opravil S, Jenuwein T, Berger SL (2006). Repression of p53 activity by Smyd2-mediated methylation. Nature.

[37] Vousden KH, Prives C (2009). Blinded by the Light: The Growing Complexity of p53. Cell.

[38] Ossovskaya VS, Mazo IA, Chernov MV, Chernova OB, Strezoska Z, Kondratov R, Stark GR, Chumakov PM, Gudkov AV (1996). Use of genetic suppressor elements to dissect distinct biological effects of separate p53 domains. Proc Natl Acad Sci U S A.

[39] Arrowsmith CH, Bountra C, Fish PV, Lee K, Schapira M (2012). Epigenetic protein families: a new frontier for drug discovery. Nature reviews Drug discovery.

[40] Barsyte-Lovejoy D, Li F, Oudhoff MJ, Tatlock JH, Dong A, Zeng H, Wu H, Freeman SA, Schapira M, Senisterra GA, Kuznetsova E, Marcellus R, Allali-Hassani A (2014). (R)-PFI-2 is a potent and selective inhibitor of SETD7 methyltransferase activity in cells. Proc Natl Acad Sci USA.

[41] Han Y, Cui J, Lu Y, Sue S, Arpaia E, Mak TW, Minden MD (2012). FCHSD2 predicts response to chemotherapy in acute myeloid leukemia patients. Leuk Res.

[42] Rhodes DR, Yu J, Shanker K, Deshpande N, Varambally R, Ghosh D, Barrette T, Pandey A, Chinnaiyan AM (2004). ONCOMINE: a cancer microarray database and integrated data-mining platform. Neoplasia.

[43] Papaemmanuil E, Gerstung M, Bullinger L, Gaidzik VI, Paschka P, Roberts ND, Potter NE, Heuser M, Thol F, Bolli N, Gundem G, Van Loo P, Martincorena I (2016). Genomic Classification and Prognosis in Acute Myeloid Leukemia. N Engl J Med.

[44] Cancer Genome Atlas Research N (2013). Genomic and epigenomic landscapes of adult de novo acute myeloid leukemia. N Engl J Med.

[45] Eppert K, Takenaka K, Lechman ER, Waldron L, Nilsson B, van Galen P, Metzeler KH, Poeppl A, Ling V, Beyene J, Canty AJ, Danska JS, Bohlander SK (2011). Stem cell gene expression programs influence clinical outcome in human leukemia. Nature medicine.

[46] Bunjobpol W, Dulloo I, Igarashi K, Concin N, Matsuo K, Sabapathy K (2014). Suppression of acetylpolyamine oxidase by selected AP-1 members regulates DNp73 abundance: mechanistic insights for overcoming DNp73-mediated resistance to chemotherapeutic drugs. Cell Death Differ.

[47] Burgess DJ, Doles J, Zender L, Xue W, Ma B, McCombie WR, Hannon GJ, Lowe SW, Hemann MT (2008). Topoisomerase levels determine chemotherapy response in vitro and in vivo. Proc Natl Acad Sci U S A.

[48] Sykes DB, Kfoury YS, Mercier FE, Wawer MJ, Law JM, Haynes MK, Lewis TA, Schajnovitz A, Jain E, Lee D, Meyer H, Pierce KA, Tolliday NJ (2016). Inhibition of Dihydroorotate Dehydrogenase Overcomes Differentiation Blockade in Acute Myeloid Leukemia. Cell.

[49] Essers MA, Trumpp A (2010). Targeting leukemic stem cells by breaking their dormancy. Molecular oncology.

[50] Sese B, Barrero MJ, Fabregat MC, Sander V, Izpisua Belmonte JC (2013). SMYD2 is induced during cell differentiation and participates in early development. Int J Dev Biol.

[51] Abu-Farha M, Lambert JP, Al-Madhoun AS, Elisma F, Skerjanc IS, Figeys D (2008). The tale of two domains: proteomics and genomics analysis of SMYD2, a new histone methyltransferase. Molecular & cellular proteomics.

[52] Saddic LA, West LE, Aslanian A, Yates JR, Rubin SM, Gozani O, Sage J (2010). Methylation of the retinoblastoma tumor suppressor by SMYD2. J Biol Chem.

[53] Zhang X, Tanaka K, Yan J, Li J, Peng D, Jiang Y, Yang Z, Barton MC, Wen H, Shi X (2013). Regulation of estrogen receptor alpha by histone methyltransferase SMYD2-mediated protein methylation. Proc Natl Acad Sci U S A.

[54] Donlin LT, Andresen C, Just S, Rudensky E, Pappas CT, Kruger M, Jacobs EY, Unger A, Zieseniss A, Dobenecker MW, Voelkel T, Chait BT, Gregorio CC (2012). Smyd2 controls cytoplasmic lysine methylation of Hsp90 and myofilament organization. Genes Dev.

[55] Abu-Farha M, Lanouette S, Elisma F, Tremblay V, Butson J, Figeys D, Couture JF (2011). Proteomic analyses of the SMYD family interactomes identify HSP90 as a novel target for SMYD2. Journal of molecular cell biology.

[56] Nakakido M, Deng Z, Suzuki T, Dohmae N, Nakamura Y, Hamamoto R (2015). Dysregulation of AKT Pathway by SMYD2-Mediated Lysine Methylation on PTEN. Neoplasia.

[57] Reynoird N, Mazur PK, Stellfeld T, Flores NM, Lofgren SM, Carlson SM, Brambilla E, Hainaut P, Kaznowska EB, Arrowsmith CH, Khatri P, Stresemann C, Gozani O (2016). Coordination of stress signals by the lysine methyltransferase SMYD2 promotes pancreatic cancer. Genes Dev.

[58] Lanouette S, Davey JA, Elisma F, Ning Z, Figeys D, Chica RA, Couture JF (2015). Discovery of substrates for a SET domain lysine methyltransferase predicted by multistate computational protein design. Structure.

[59] Olsen JB, Cao XJ, Han B, Chen LH, Horvath A, Richardson TI, Campbell RM, Garcia BA, Nguyen H (2016). Quantitative Profiling of the Activity of Protein Lysine Methyltransferase SMYD2 Using SILAC-Based Proteomics. Molecular & cellular proteomics.

[60] Ahmed H, Duan S, Arrowsmith CH, Barsyte-Lovejoy D, Schapira M (2016). An Integrative Proteomic Approach Identifies Novel Cellular SMYD2 Substrates. Journal of proteome research.

[61] Cheng T, Rodrigues N, Shen H, Yang Y, Dombkowski D, Sykes M, Scadden DT (2000). Hematopoietic stem cell quiescence maintained by p21cip1/waf1. Science.

[62] el-Deiry WS, Tokino T, Velculescu VE, Levy DB, Parsons R, Trent JM, Lin D, Mercer WE, Kinzler KW, Vogelstein B (1993). WAF1, a potential mediator of p53 tumor suppression. Cell.

[63] El-Deiry WS, el-Deiry WS, Tokino T Fau-Velculescu VE, Velculescu Ve Fau-Levy DB, Levy Db Fau-Parsons R, Parsons R Fau-Trent JM, Trent Jm Fau-Lin D, Lin D Fau-Mercer WE, Mercer We Fau-Kinzler KW, Kinzler Kw Fau-Vogelstein B, Vogelstein B p21(WAF1) Mediates Cell-Cycle Inhibition, Relevant to Cancer Suppression and Therapy WAF1, a potential mediator of p53 tumor suppression.

[64] Seoane J, Le HV, Shen L, Anderson SA, Massague J (2004). Integration of Smad and forkhead pathways in the control of neuroepithelial and glioblastoma cell proliferation. Cell.

[65] Lezina L, Aksenova V, Ivanova T, Purmessur N, Antonov AV, Tentler D, Fedorova O, Garabadgiu AV, Talianidis I, Melino G, Barlev NA (2014). KMTase Set7/9 is a critical regulator of E2F1 activity upon genotoxic stress. Cell Death Differ.

[66] Jost CA, Marin MC, Kaelin WG (1997). p73 is a simian [correction of human] p53-related protein that can induce apoptosis. Nature.

[67] Zhang X, Huang Y, Shi X (2015). Emerging roles of lysine methylation on non-histone proteins. Cell Mol Life Sci.

[68] Marouco D, Garabadgiu AV, Melino G, Barlev NA (2013). Lysine-specific modifications of p53: a matter of life and death?. Oncotarget.

[69] Pires-Luis AS, Vieira-Coimbra M, Vieira FQ, Costa-Pinheiro P, Silva-Santos R, Dias PC, Antunes L, Lobo F, Oliveira J, Goncalves CS, Costa BM, Henrique R, Jeronimo C (2015). Expression of histone methyltransferases as novel biomarkers for renal cell tumor diagnosis and prognostication. Epigenetics.

[70] Oliveira-Santos W, Rabello DA, Lucena-Araujo AR, de Oliveira FM, Rego EM, Pittella Silva F, Saldanha-Araujo F (2016). Residual expression of SMYD2 and SMYD3 is associated with the acquisition of complex karyotype in chronic lymphocytic leukemia. Tumour biology.

[71] Komatsu S, Ichikawa D, Hirajima S, Nagata H, Nishimura Y, Kawaguchi T, Miyamae M, Okajima W, Ohashi T, Konishi H, Shiozaki A, Fujiwara H, Okamoto K (2015). Overexpression of SMYD2 contributes to malignant outcome in gastric cancer. Br J Cancer.

[72] Sakamoto LH, Andrade RV, Felipe MS, Motoyama AB, Pittella Silva F (2014). SMYD2 is highly expressed in pediatric acute lymphoblastic leukemia and constitutes a bad prognostic factor. Leuk Res.

[73] Ohtomo-Oda R, Komatsu S, Mori T, Sekine S, Hirajima S, Yoshimoto S, Kanai Y, Otsuji E, Ikeda E, Tsuda H (2016). SMYD2 overexpression is associated with tumor cell proliferation and a worse outcome in human papillomavirus-unrelated nonmultiple head and neck carcinomas. Human pathology.

[74] Gavosto F (1969). Cell kinetics in acute lymphoblastic leukaemia. Br J Haematol.

[75] Ishikawa F, Yoshida S, Saito Y, Hijikata A, Kitamura H, Tanaka S, Nakamura R, Tanaka T, Tomiyama H, Saito N, Fukata M, Miyamoto T, Lyons B (2007). Chemotherapy-resistant human AML stem cells home to and engraft within the bone-marrow endosteal region. Nat Biotechnol.

[76] Bagislar S, Sabo A, Kress TR, Doni M, Nicoli P, Campaner S, Amati B (2016). Smyd2 is a Myc-regulated gene critical for MLL-AF9 induced leukemogenesis. Oncotarget.

[77] Moffat J, Grueneberg DA, Yang X, Kim SY, Kloepfer AM, Hinkle G, Piqani B, Eisenhaure TM, Luo B, Grenier JK, Carpenter AE, Foo SY, Stewart SA (2006). A lentiviral RNAi library for human and mouse genes applied to an arrayed viral high-content screen. Cell.

[78] Armstrong SA, Staunton JE, Silverman LB, Pieters R, den Boer ML, Minden MD, Sallan SE, Lander ES, Golub TR, Korsmeyer SJ (2002). MLL translocations specify a distinct gene expression profile that distinguishes a unique leukemia. Nat Genet.

