# Supplementary material for: SMYD2 lysine methyltransferase regulates leukemia cell growth and regeneration after genotoxic stress
Source: Oncotarget. 2017 Feb 6;8(10):16712–27. doi: 10.18632/oncotarget.15147 (PMC5369996; doi:10.18632/oncotarget.15147)
Supplement: Supplementary file 1 [file oncotarget-08-16712-s001.pdf]

SMYD2 lysine methyltransferase regulates leukemia cell growth and regeneration after genotoxic stress

Supplementary Materials

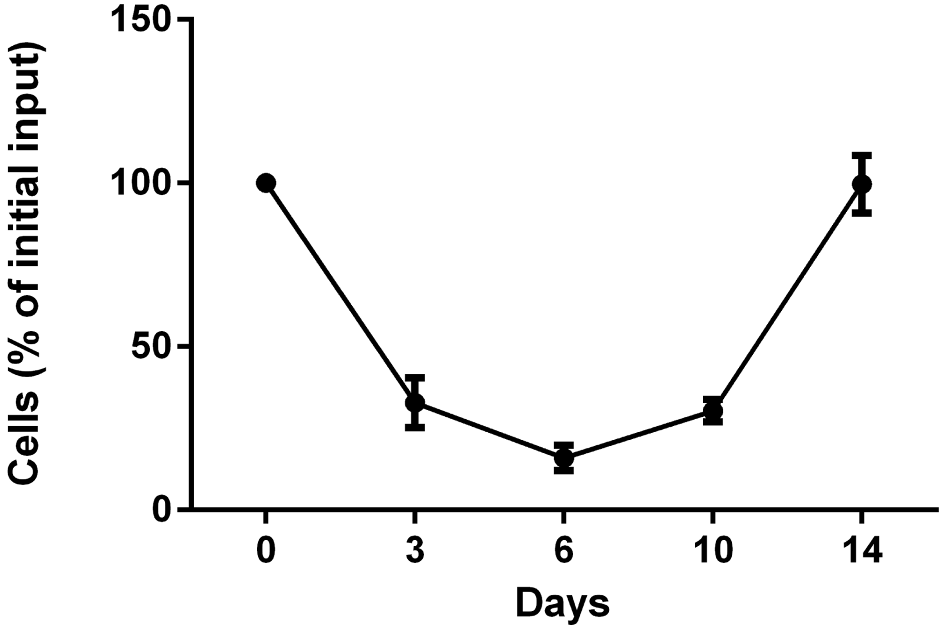

Supplementary Figure 1: TEX proliferation after 4Gy IR. Cells expansion was calculated by viable counting (*n* = 4).

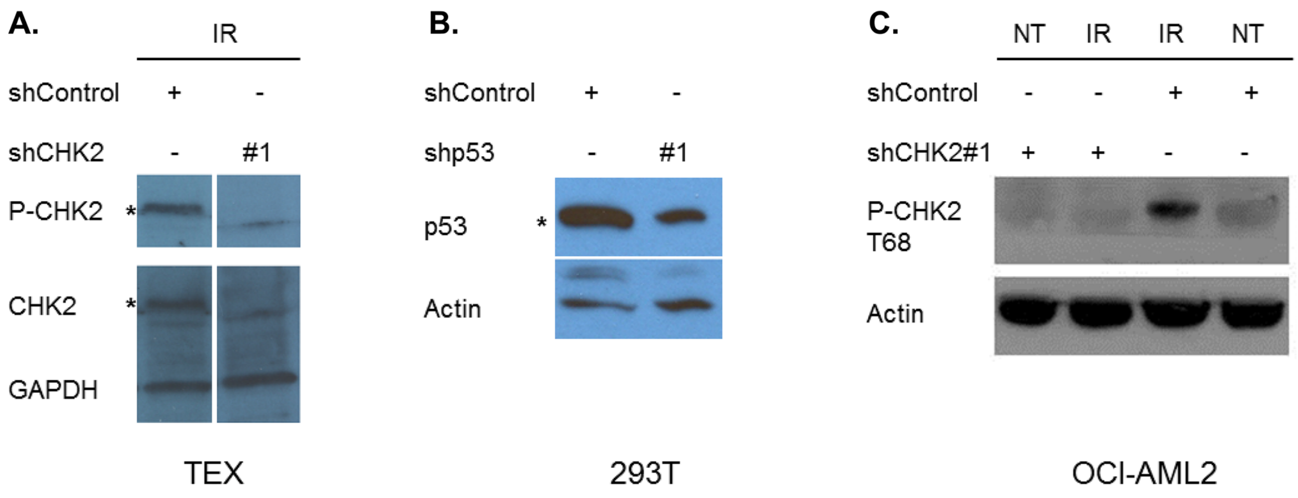

Supplementary Figure 2: Validation of CHK2 and p53 directed shRNAs used in the study by Western blot analysis. Levels of Phospho-CHK2 and CHK2 in TEX and OCI-AML2 cells were measure after induction with 4 Gy of irradiation (**A**, **C**). 293T cells expressing abundant p53 levels were used to validate shp53 (**B**). Actin and GAPDH levels represent a loading control.

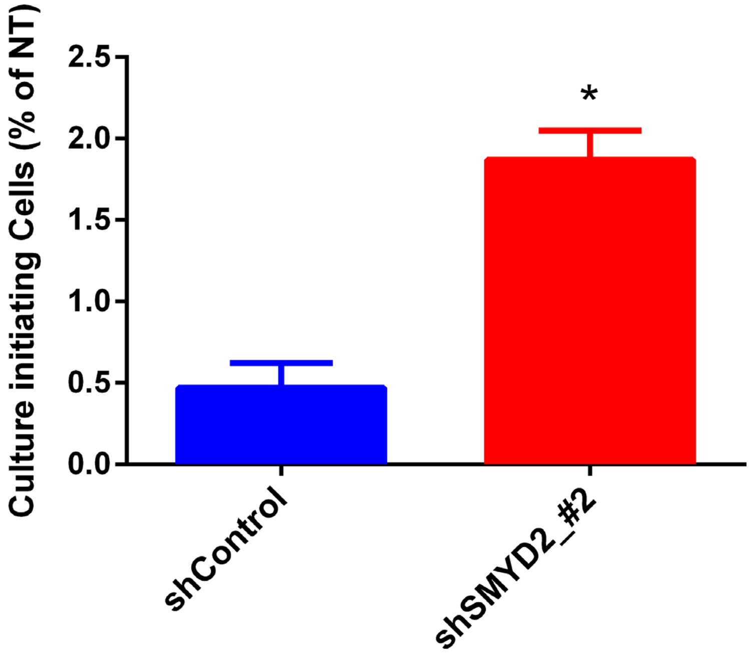

Supplementary Figure 3: TEX cells infected with shControl or SMYD2 knockdown shRNAs and selected with puromycin were treated with Etoposide (5 µM for 24 hrs) and plated in 96-well plate at different cell/well doses (NT: 2, 5, 10, 30 cells/well; Etoposide: 20, 50, 100, 200 cells/well). 16 days post incubation the number of wells with colonies per each cell dose was recorded and used to calculate culture initiating cell frequency with L-Calc™ Limiting Dilution Software from STEMCELL Technologies, Inc (Canada). ($n = 3$, $*p < 0.02$).

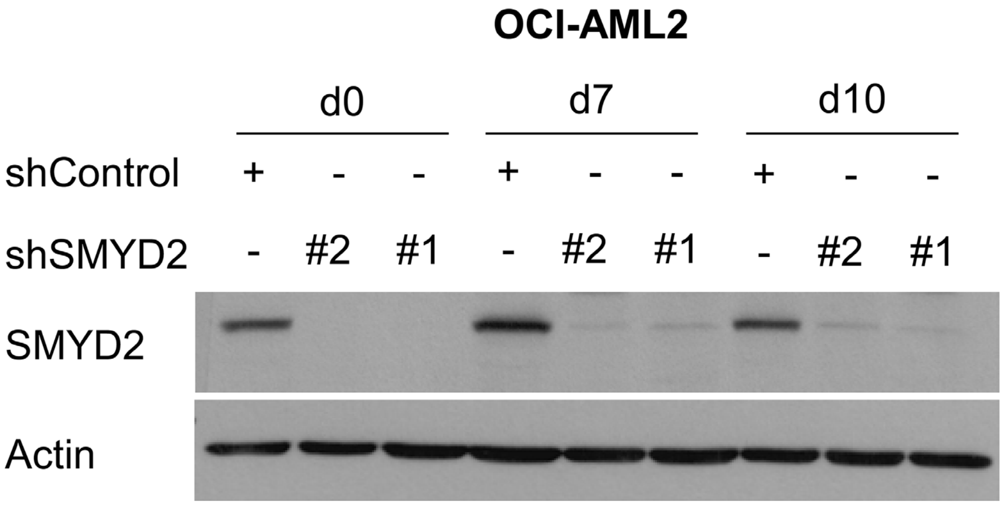

Supplementary Figure 4: Western blot analysis of SMYD2 levels in OCI-AML2 cells expressing shSMYD2 at the different time points post 10 infection. Actin levels represent a loading control (*n* = 3).

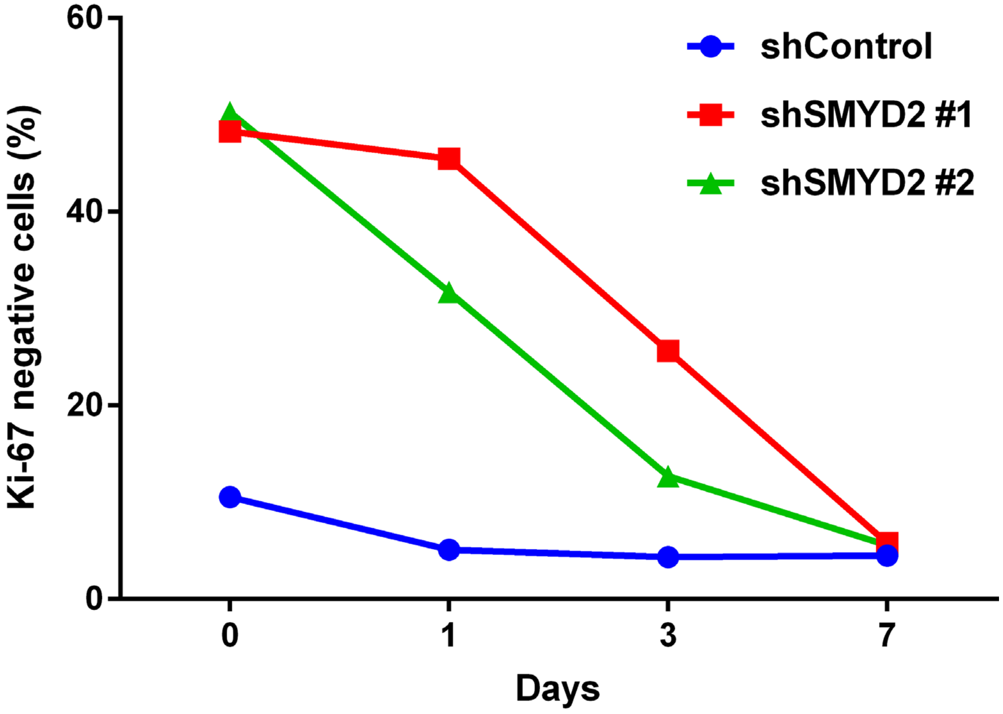

Supplementary Figure 5: Kinetic analysis of Ki-67 negative cells in shControl and shSMYD2 expressing OCI-AML2 cells at 0, 1, 3 and 7 days post selection using Ki-67/DAPI based flow cytometry analysis.

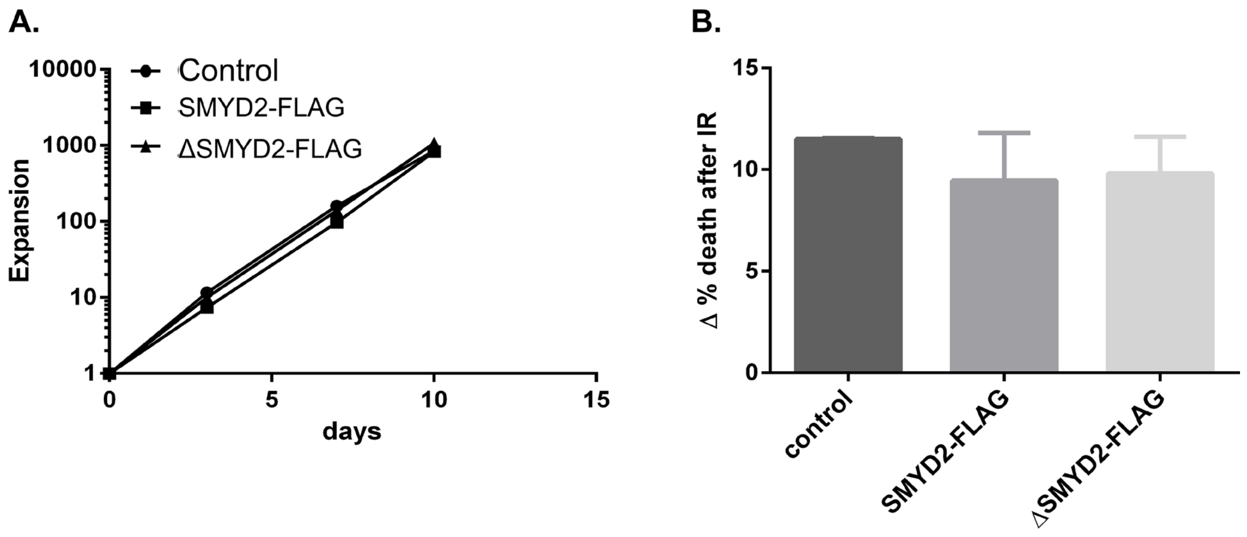

Supplementary Figure 6: SMYD2 over expression (OE) does not affect cells growth and IR response. (**A**) Expansion of OCI-AML2 cells infected with OSMYD2-FLAG or SMYD2-ΔNHSC/ΔGEEV-FLAG dead enzyme. Cells expansion was calculated by viable counting (*n* = 2). (**B**) Annexin V/Sytox blue flow cytometry analysis of OCI-AML2 cells expressing SMYD2 constructs upon 4 Gy IR.

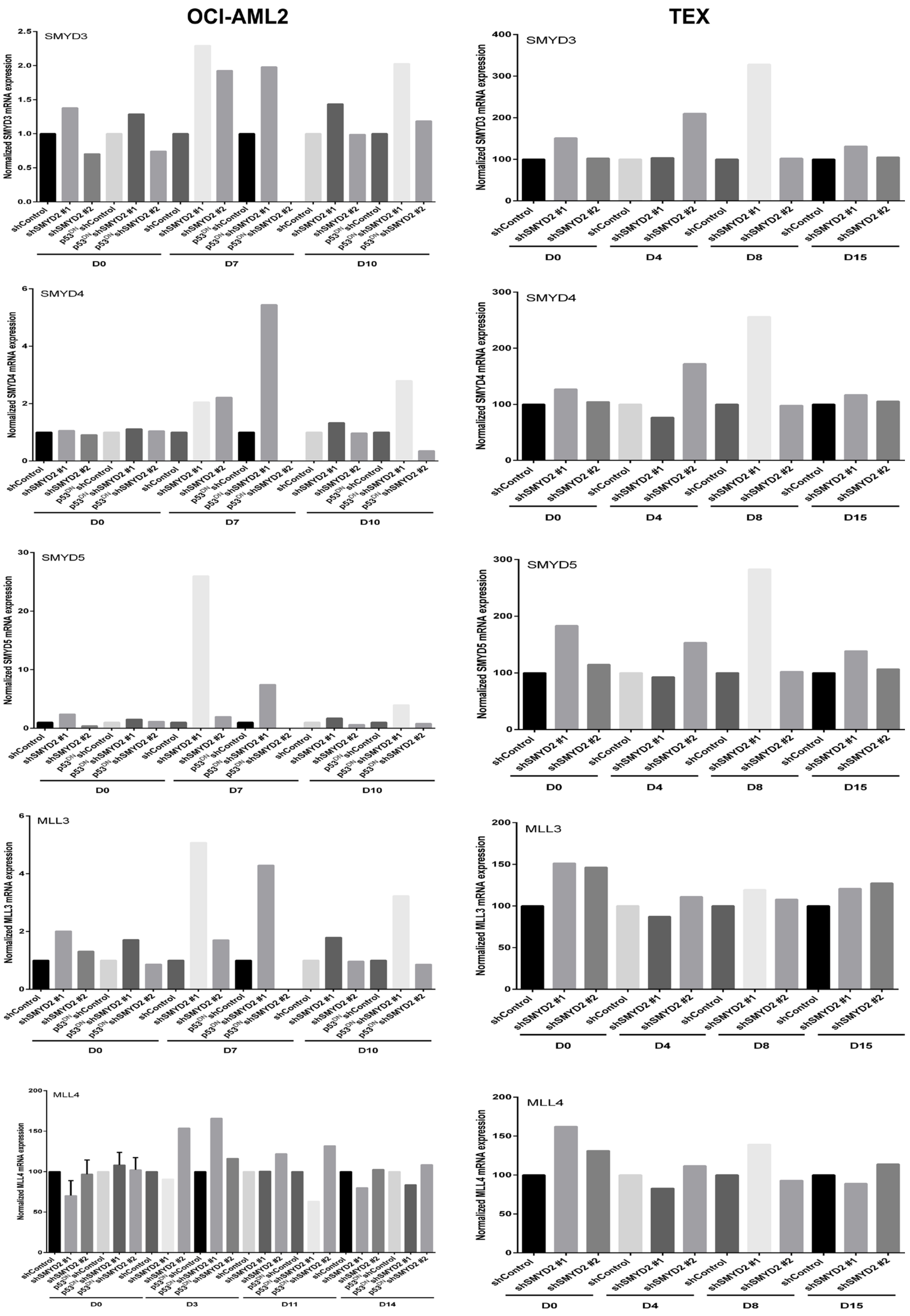

Supplementary Figure 7: KMTs expression levels in OCI-AML2 and TEX cells. SMYD3, SMYD4, SMYD5, MLL3 and MLL4 mRNAs were determined using qRT-PCR in OCI-AML2 and TEX cells expressing indicated lenticonstructs. Day after infection is indicated under the line (*n* = 1–2).

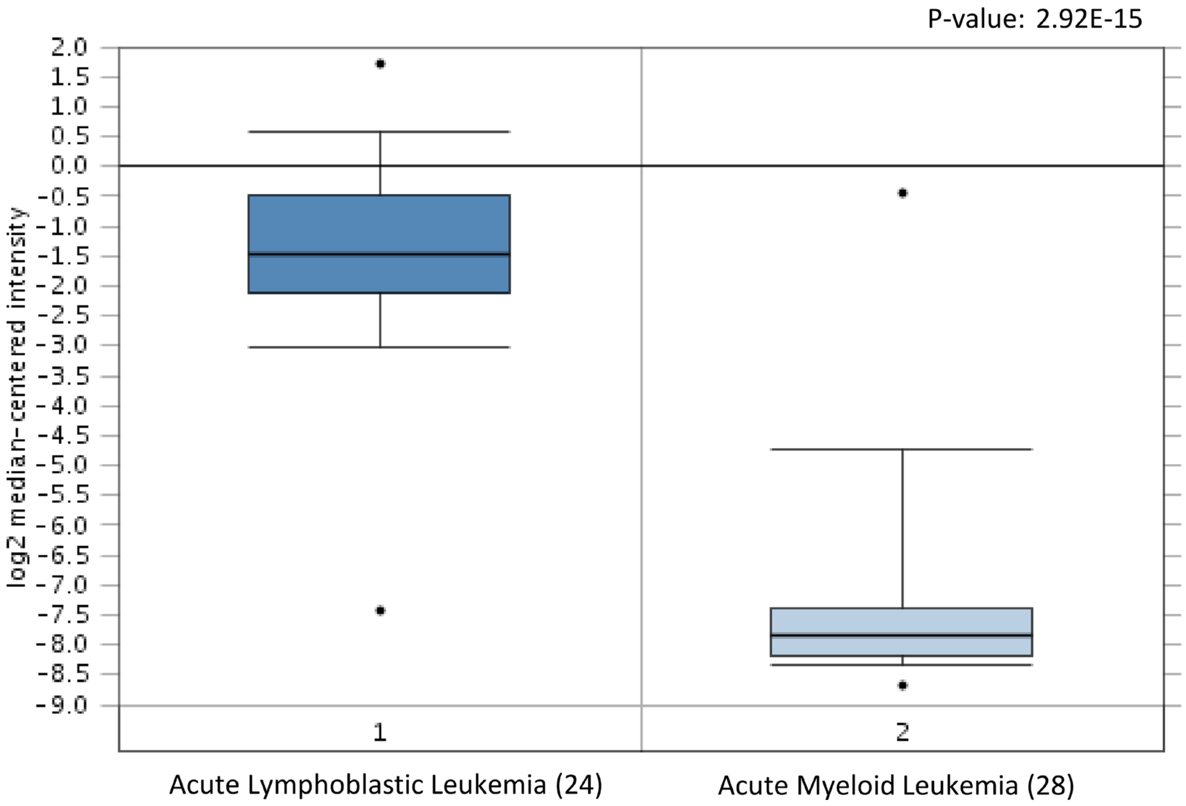

Supplementary Figure 8: Differential expression of SMYD2 in Acute Lymphoblastic Leukemia and Acute Myeloid Leukemia. Depicted is the relative expression of SMYD2 according to leukemia types based on the Armstrong et al. leukemia dataset [78] as obtained from the ONCOMINE database. The number of patients in each group is indicated in parentheses.

Supplementary Table 1: Inhibition of SET7/9 attenuates shSMYD2 expressing cells expansion

		day 0	day 6	day 12
Exp1	shControl_DMSO	1	10.4	19.2
	shControl_R-PFI 10 µM	1	5.6	7.6
	shSMYD2_DMSO	1	1.6	2
	shSMYD2_R-PFI 10 µM	1	0.4	0.01
Exp2	shControl_DMSO	1	8.55	125.3
	shControl_R-PFI 10 µM	1	10.4	113.8
	shSMYD2_DMSO	1	9.45	60.5
	shSMYD2_R-PFI 10 µM	1	5.9	16.6
Exp3	shControl_DMSO	1	52	756.8
	shControl_R-PFI 10 µM	1	118.4	629.0
	shSMYD2_DMSO	1	11.4	299.2
	shSMYD2_R-PFI 10 µM	1	15.12	127.0

Cell expansion was calculated by viable counting at days 6 and 12 after infection. Raw data of 3 experiments is presented. shControl or shSMYD2 cells were grown in the presence of DMSO or 10 µM R-PFI (SET7/9 inhibitor).
